# A cyclical marker system enables indefinite series of oligonucleotide-directed gene editing in *Chlamydomonas reinhardtii*

**DOI:** 10.1093/plphys/kiae427

**Published:** 2024-08-23

**Authors:** Ian L Ross, Hong Phuong Le, Sabar Budiman, Dake Xiong, Fritz Hemker, Elizabeth A Millen, Melanie Oey, Ben Hankamer

**Affiliations:** Institute for Molecular Bioscience (IMB), The University of Queensland, Brisbane, QLD 4072, Australia; Institute for Molecular Bioscience (IMB), The University of Queensland, Brisbane, QLD 4072, Australia; Institute for Molecular Bioscience (IMB), The University of Queensland, Brisbane, QLD 4072, Australia; Institute for Molecular Bioscience (IMB), The University of Queensland, Brisbane, QLD 4072, Australia; Institute for Molecular Bioscience (IMB), The University of Queensland, Brisbane, QLD 4072, Australia; Institute for Molecular Bioscience (IMB), The University of Queensland, Brisbane, QLD 4072, Australia; Institute for Molecular Bioscience (IMB), The University of Queensland, Brisbane, QLD 4072, Australia; Institute for Molecular Bioscience (IMB), The University of Queensland, Brisbane, QLD 4072, Australia

## Abstract

CRISPR/Cas9 gene editing in the model green alga *Chlamydomonas reinhardtii* relies on the use of selective marker genes to enrich for nonselectable target mutations. This becomes challenging when many sequential modifications are required in a single-cell line, as useful markers are limited. Here, we demonstrate a cyclical selection process which only requires a single marker gene to identify an almost infinite sequential series of CRISPR-based target gene modifications. We used the *NIA1* (*Nit1, NR*; nitrate reductase) gene as the selectable marker in this study. In the forward stage of the cycle, a stop codon was engineered into the *NIA1* gene at the CRISPR target location. Cells retaining the wild-type *NIA1* gene were killed by chlorate, while *NIA1* knockout mutants survived. In the reverse phase of the cycle, the stop codon engineered into the *NIA1* gene during the forward phase was edited back to the wild-type sequence. Using nitrate as the sole nitrogen source, only the reverted wild-type cells survived. By using CRISPR to specifically deactivate and reactivate the *NIA1* gene, a marker system was established that flipped back and forth between chlorate- and auxotrophic (nitrate)-based selection. This provided a scarless cyclical marker system that enabled an indefinite series of CRISPR edits in other, nonselectable genes. We demonstrate that this “Sequential CRISPR via Recycling Endogenous Auxotrophic Markers (SCREAM)” technology enables an essentially limitless series of genetic modifications to be introduced into a single-cell lineage of *C. reinhardtii* in a fast and efficient manner to complete complex genetic engineering.

## Teaser

Unlimited cycles of gene edits in *Chlamydomonas* using a recyclable selectable marker gene enable complex edited strains in a wild-type background.

## Introduction

Genetically editing specific targeted changes into the genome of living organisms is central to modern biology, and the discovery of Clustered Regularly Interspaced Short Palindromic Repeat (CRISPR) approaches has greatly accelerated this editing ability. CRISPR employs ribonuclear protein (RNP) complexes consisting of short targeting guide RNAs and CRISPR-associated proteins (e.g. Cas9; Charpentier and Doudna (2013)). CRISPR-mediated gene editing has many biological applications, including in algal biology, where biotechnological solutions are sought to advance renewable energy (Stephens et al. 2010), food and high value products, and bio/nano materials (Karan et al. 2019; Ross et al. 2021). In some microalgal species, CRISPR/Cas9 editing has been straightforward and successful, including *Nannochloropsis* (Wang et al. 2016) and *Phaeodactylum* (Nymark et al. 2016); in others, such as *Chlamydomonas,* it has been challenging, with low rates of transformation compared with other species, especially when transient expression of Cas9 and gRNAs were employed (Jiang et al. 2014; Guzmán-Zapata et al. 2019).

*Chlamydomonas* is an important algal genetic model system, especially in photosynthesis and cilia biology (Harris 2009). The quality of the *Chlamydomonas* genome sequence, the detailed understanding of its biology in comparison to other algal species, and the existence of large collections of well-characterized mutants make it a preeminent model organism for genetic modification for biotechnological and renewable fuel-related applications, including the expression of recombinant proteins (Tran et al. 2013; Rasala and Mayfield 2015; Scranton et al. 2015; Zedler et al. 2016; Ramos-Martinez et al. 2017; Baier et al. 2018).

Pioneering CRISPR/Cas9 editing work in *Chlamydomonas* by Shin et al. (2016) and Baek et al. (2016) employed transfected RNPs. Although the expression of Cas9 and guide RNAs from plasmids or genomic constructs has been achieved (Jiang et al. 2014; Greiner et al. 2017; Jiang and Weeks 2017; Guzman-Zapata et al. 2019; Park et al. 2020), the RNP approach is simple to employ, efficient, and has been widely used (Ferenczi et al. 2017; Greiner et al. 2017; Shamoto et al. 2018; Angstenberger et al. 2020; Cazzaniga et al. 2020; Dhokane et al. 2020; Kang et al. 2020; Kim et al. 2020; Picariello et al. 2020; Akella et al. 2021). Due to the relatively low CRISPR modification rates in *Chlamydomonas*, this approach typically employs marker systems, such as antibiotic selection genes, to enrich transformed populations prior to screening for gene edited cells. In *Chlamydomonas*, foreign transgenes such as selectable marker genes are typically inserted by nonhomologous or theta-mediated end joining (NHEJ/TMEJ; Ferenczi et al. (2021); Sizova et al. (2021)) at the site of a double-strand break, such as that produced at the target site by Cas9 (Baek et al. 2016; Shin et al. 2016). Importantly, Ferenczi et al. (2017) showed that single-stranded oligonucleotides could be used for CRISPR-directed homologous recombination, with increased rates of successful modification. This CRISPR/ssODN strategy has also been reported in other organisms (Hwang et al. 2013; Auer and Del Bene 2014; Bialk et al. 2015) while CRISPR was recently used for gRNA-directed targeted insertion of large inserts in *Chlamydomonas*, up to 6 kb (Kim et al. 2020).

The most successful and widely used selection markers in *Chlamydomonas* are exogenous antibiotic selection genes including aminoglycosidase adenyltransferase (*aadA;* Cerutti et al. (1997)), aminoglycoside-O-phosphotransferase VIII (*aphVIII;* Sizova et al. (1996)) and aminoglycoside phosphotransferase 7 (*aph7;* Berthold et al. (2002)) which, when transfected, confer resistance to spectinomycin/streptomycin, paromomycin and hygromycin, respectively. Endogenous nonessential genes can also be used as *counter-selectable* markers, with mutants displaying resistance to toxic xenobiotics that kill wild-type cells. Examples include mutations in adenine phosphoribosyltransferase (*APRT;* Schaff (1994)), tryptophan synthase beta (*MAA7;* Palombella and Dutcher (1998)), protoporphyrinogen oxidase (*PPX1*; Kovar et al. (2002)) and acetolactate synthase (*ALS1;* Akella et al. (2021)).

Finally, auxotrophic strains including mutants in argininosuccinate lyase (*ARG7* Purton and Rochaix (1995)), quinolinate synthetase A (*THI10;* Ferris (1995)), hydroxyethylthiazole kinase (*NIC7;* Ferris (1995)), nitrate reductase (*NIA1;* Kindle et al. (1989)), oxygen evolution enhancer protein 1 (*PSBO;* Mayfield and Kindle (1990)) and spermidine synthase (*SPD1;* Freudenberg et al. (2022)) genes have been employed as selectable markers. As with antibiotic marker cassettes, transfection with a complementing gene usually involves random insertion creating a genetic “scar”. In a recent advance, Akella et al. (2021) showed that by CRISPR targeting a selectable host gene along with the gene of interest, the use of co-transfected marker gene DNA could be avoided, leading to precise and scarless editing of the target gene.

Rapid, sequential editing to produce complex phenotypes is the next key challenge of CRISPR editing in *Chlamydomonas.* This would eliminate the need to use multiple markers or backcross single mutants to create multiply edited cell lines, for example to develop multiple simultaneous knockouts (e.g. to analyse complex traits), to introduce multiple point mutations (e.g. metabolic engineering) or to identify key amino acids in a target protein. This capability facilitates the creation of highly genome-modified algal cell lines to advance fundamental science and industrial production. Importantly, existing cell lines resulting from previous engineered genome changes could be rapidly modified to create new gene variants without the need to retrace engineering steps.

Here we report the SCREAM (*Sequential CRISPR via Recycling Endogenous Auxotrophic Markers*) strategy; an efficient, convenient, reusable, scarless, fully reversible marker strategy which enables sequential addition of genetic modifications, without introducing fundamental changes to the original genotype. Using the same marker for all sequential target gene modifications establishes a standardized CRISPR technique that can be used to modify any set of target genes. We also sought a system that could be applied to a wide range of preexisting strains and would not require the inclusion of a specific cassette or GMO construct into the cell line of interest.

Importantly, by regenerating the wild-type selectable marker, SCREAM allows mutation of nonselectable target genes without the insertion of foreign marker DNA. The resulting organism is non-GMO in many global jurisdictions, with important commercial implications.

The SCREAM strategy requires a marker gene that enables both forward- and reverse-selection. It uses single-stranded oligodeoxynucleotide (ssODN) based editing to introduce (and then to reverse) a precise mutation (e.g. a stop codon) that flips the selection phenotype back and forth. This regenerates the original gene after each two-step cycle, and incorporates useful modifications that simplify screening.

Here, the nitrate reductase (*NR; NIA1; Nit1*) gene was chosen as the selection system (Fernández and Matagne 1984; Fernández et al. 1989). *NIA1* enables assimilation of nitrate as the sole source of nitrogen. Mutations in *NIA1* prevent *Chlamydomonas* from growing on nitrate, but *NIA1* mutants can be positively selected on media containing sodium or potassium chlorate (ClO_3_^−^) since the loss of NR activity confers resistance to chlorate (Prieto and Fernández 1993). Both forward (chlorate) and reverse (nitrate) selection strategies are consequently encoded by the one gene, *NIA1*. The single-copy nitrate reductase gene *NIA1* has long been studied in the context of nitrate assimilation (Sanz-Luque et al. 2015) and was also the first gene used for nuclear transformation (and co-transformation) of *Chlamydomonas* (Fernández and Matagne 1984; Kindle 1990; Blankenship and Kindle 1992).

In forward SCREAM, we employed an ssODN with Cas9 CRISPR RNPs to create a precise stop codon in the *NIA1* gene. Chlorate selection was used to enrich for *NIA1^−^* clones. For the reverse step (ssODN-mediated *NIA1* repair), we employed nitrate auxotrophic selection to identify genome-edited clones in which repair of *NIA1* has occurred.

This approach enabled the use of a uniform selection assay strategy for any CRISPR-based target gene modification, provided a scarless marker and enables a limitless cyclical series of gene editing events. The widespread presence of nitrate reductase in other algal species and in land plants suggests that SCREAM using nitrate reductase offers a wide array of opportunities for plant biotechnology. However, other endogenous dual selectable marker genes could also be employed.

## Results and discussion

### Strategy: *NIA1* as an endogenous marker for SCREAM experiments

**Figure 1.**
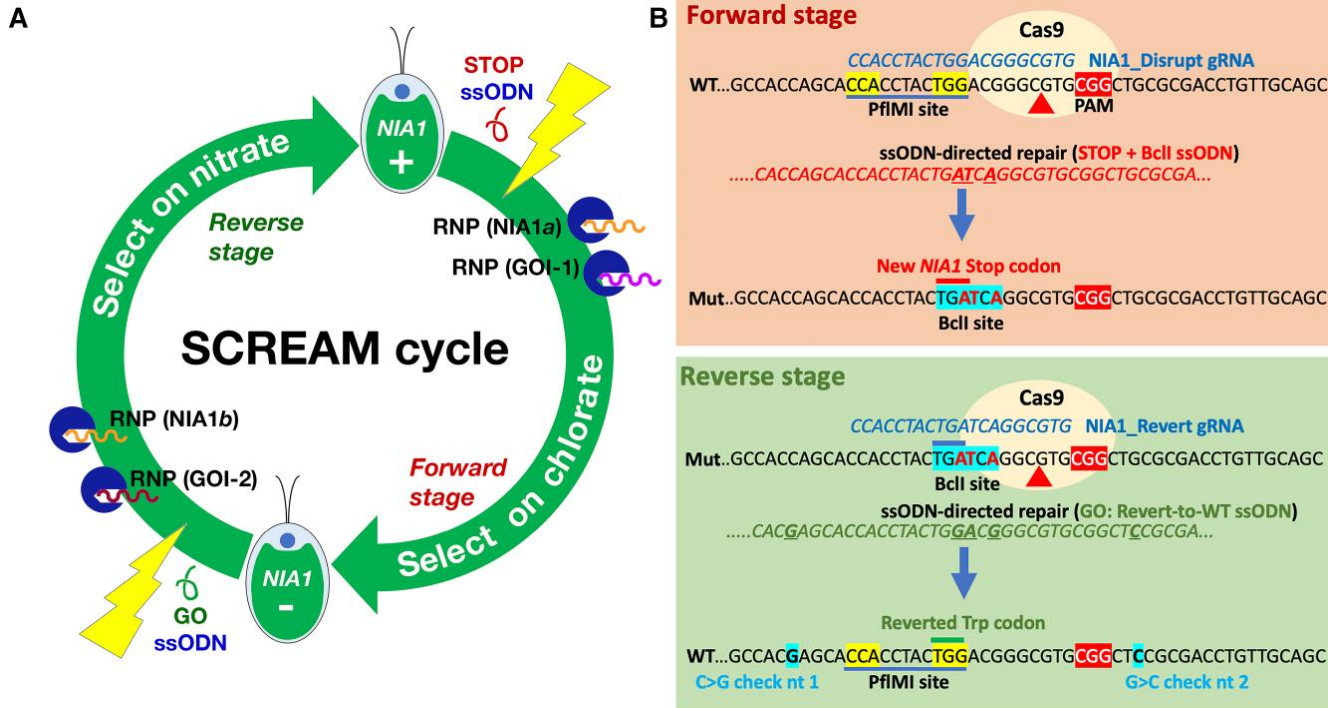
Schematic for sequential CRISPR via recycling endogenous auxotrophic markers (SCREAM). **A)** Illustration of basic SCREAM strategy. During the forward stage, *NIA1^+^* cells (top) are electroporated with a *NIA1* “STOP” codon-containing single stranded oligonucleotide (ssODN), as well as ribonucleoproteins (RNPs) comprised of Cas9 (circles) bound to guide RNAs (gRNAs) directed against both the wild-type (WT) *NIA1* gene (RNP NIA1*a*) and the first gene of interest (GOI-1). After selection for *NIA1* cells on chlorate, cells containing both the *NIA1* stop codon (detected by *Bcl*I digestion of PCR amplicon) and the mutated GOI-1 (identified by gene specific PCR) are identified. In the reverse stage, the gRNA (NIA1*b*) targets the mutated *NIA1* and the *NIA1* “GO” (i.e. revert to WT) ssODN restores the wild-type *NIA1* gene while the additional gRNA (GOI-2) targets the second gene of interest. The resultant edited *NIA1^+^* cells are able to grow on nitrate and can be selected on nitrate plates. Each cycle thus allows at least two genes of interest to be edited. For simplicity, other variants described (e.g. use of target gene ssODNs or dual targeting RNAs) are not included in this figure. **B)** Detail of the ssODNs employed for homology-directed repair (HDR). The *NIA1* Cas9 guide RNAs and the protospacer adjacent motif (highlighted, with white text) are shown, with the most likely cleavage site (arrow). In the forward stage *NIA1* inactivation step (upper panel), the NIA1_STOP_BclI ssODN inserts a stop codon (TGG > TGA) that truncates *NIA1* translation in exon 2. It also eliminates a wild-type *Pfl*MI site (highlighted in WT) which relies on the TGG codon and installs a *Bcl*I site (TGATCA; highlighted in mutant). Nuclease digestion of PCR amplicons with *Bcl*I and/or *Pfl*MI thus enables rapid identification of *NIA1^−^* PCR amplicons likely to possess the ssODN sequence. In the reverse stage *NIA1* restoration step (lower panel), the gRNA targets the specifically altered *NIA1* gene. The NIA1_Revert-to-WT ssODN reverts this altered site back to the wild-type sequence, restoring NR activity and nitrate-competent growth, ready to start the next round of alteration. The *Pfl*MI site is also restored and the *Bcl*I site is lost. For this work, two silent mutations 5′ and 3′ to the gRNA site (bold; “check nt” 1 and 2) allowed confirmation that the ssODN has been used as a template. These would not normally be required and were included only for demonstration that HDR has occurred.

The *NIA1*-based gene editing strategy (Fig. 1A) employs guide RNAs (gRNA) assembled with Cas9 to generate target-specific ribonuclear protein (RNP) complexes. In the forward phase of SCREAM, guide RNAs are simultaneously directed at *NIA1* and the target gene(s) of interest. These are electroporated, with single stranded oligonucleotides (ssODNs) directed at *NIA1* (and, if desired, the target gene also) into *NIA1^+^ Chlamydomonas* cells. The “NIA1_STOP_BclI” ssODN contains mutations to introduce both a stop codon into *NIA1* (exon 2), and a new restriction enzyme (RE) site (*Bcl*I), to replace the natural restriction enzyme site (*Pfl*MI) in the original *NIA1* gene.

In the reverse SCREAM phase (i.e. the “revert to wild-type” experiment, Fig. 1A), the wild-type *NIA1* sequence is restored, enabling growth on nitrate. The gRNA for reversion targets the introduced stop codon and *Bcl*I site, while the corresponding NIA1_Revert-to-WT” ssODN eliminates the *Bcl*I site and re-establishes the wt gene sequence (including the wt *Pfl*MI restriction site). Additional gRNAs are simultaneously electroporated to mutate a second target gene(s) of interest. Consequently, each SCREAM cycle generates at least two new sequential target gene mutations, making sequential editing fast and efficient.

### *NIA1* deletion using CRISPR/Cas9

To validate the SCREAM process, we first confirmed that our chosen gRNAs (Table 1) successfully directed *NIA1* gene editing (Supplementary Appendix S1; Supplementary Fig. S1, A to D) without the use of ssODNs. For this we employed the *NIA1^+^* CC-1883 strain (CC-1883) derived from the basic 21 gr wild-type *NIA1^+^ Nit2^+^ Chlamydomonas reinhardtii* Sager strain (now designated CC-1690) which can grow on nitrate, with electroporation conditions initially calibrated using plasmid transformation (Supplementary Fig. S2).

**Table 1.** Guide RNAs used for CRISPR/Cas9 gene editing

Gene	gRNA name	Target sequence 5′-3′
*NIA1*	NIA1_Disrupt	CCACCUACUGGACGGGCGUG
*NIA1*	NIA1_Revert	CCACCUACUGAUCAGGCGUG
*LHCBM2*	L2-Ex1	UCCGUGCGCCCCACCGUCAG
*LHCBM1*	L1-Ex1	GGUCGAGCGGCGAGACACCG
*LHCBM1*	L1-Ex3	GGUGCUCAGAUCUUCCAGGA
*LHCBM3*	L3-Ex3	GGGGCCGUAGAACUCAAUGC
*LHCBM3*	L3-Ex4	GUAGGCGGGGGUGGCAUUCU
*LHCBM9*	L9-Ex2	CUCGAUACCAGCGCCCUUGG
*LHCBM9*	L9-Ex3	CAGAAGAACGGUGUCCAGUU
*STM6*	STM6-Ex1A	GAACGCCGGGAGUCGCCUGC
*STM6*	STM6-Ex1B	CGAUACCCCAAUAGACAGAA
*APRT*	APRT-Ex1	UGCUGUACUGGAACGCCUGG
*APRT*	APRT-Ex3	GCACAACGCGUUGCCCGGGC
*LHCA2*	Lhca2-Ex1	ACCCGCCCUAUGUGGUACCC
*LHCA2*	Lhca2-Ex2	UGAACAAGGACAACCUGAAG
*LHCA9*	Lhca9-Ex1	GAUGAUCGCUGCUAAGAGCC
*LHCA9*	Lhca9-Ex2	ACUUGAGGCGGCCCUCGUCC

Following RNP electroporation and cell recovery, selection on chlorate resulted in 871 resistant colonies. After further selecting for clones unable to grow on nitrate (i.e. *NIA1*^−^), PCR amplification of the *NIA1* locus, and in vitro Cas9 digestion of the amplicon (to identify loss of the gRNA site), 23 amplicons were sequenced. All 23 candidate mutants contained indels (Supplementary Fig. S1D) which disrupted *NIA1* structure. Most (82%) were short deletions, in line with previous reports (Baek et al. 2016; Shin et al. 2016). Despite minimal sequence changes in some mutants, all grew poorly on nitrate suggesting that even small changes in the moco binding site (exon 2) disrupted *NIA1* function. Recently, Kim et al. (2021) also showed that the *NIA1* gene was amenable to CRISPR modification in *Chlorella*.

### Specific ssODN directed *NIA1* mutation: forward SCREAM stage

**Figure 2.**
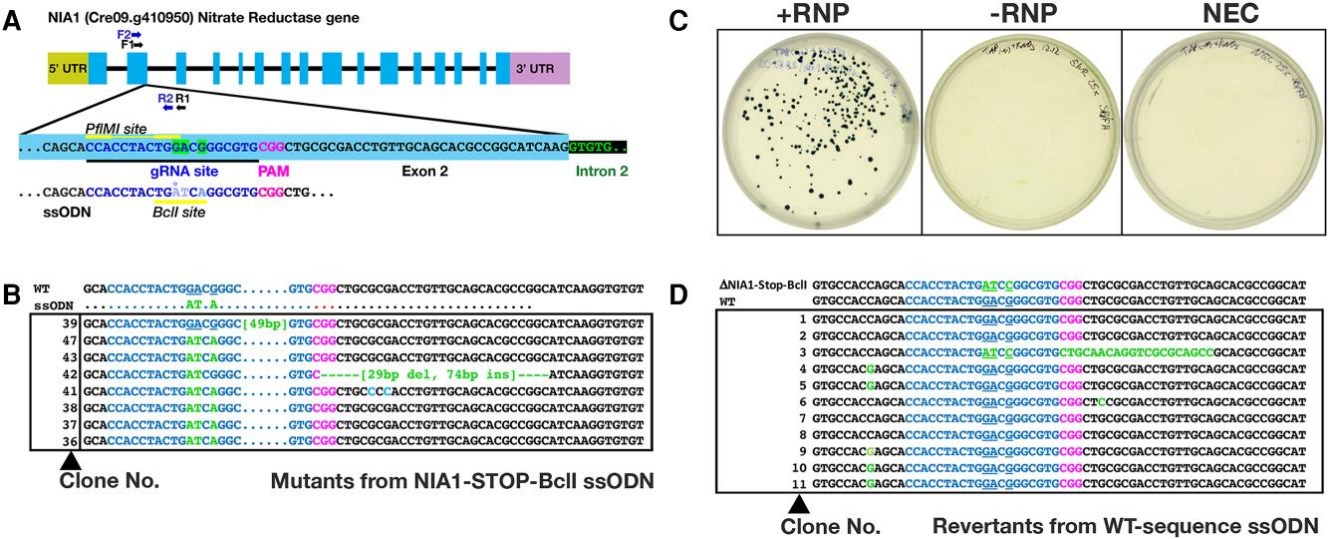
Single-stranded oligonucleotide (ssODN)-mediated *NIA1* mutation and reversion. **A)** Diagram of the *NIA1* gene structure (top; exons as blocks, narrow line for intronic regions) and the DNA sequence within the expanded Exon 2:Intron 2 region (bottom) including the guide RNA (gRNA) target site (underlined), the protospacer adjacent motif (PAM) and the native *Pfl*MI site (CCA *N_5_* TGG, lines above sequence). Within the gRNA site, bases altered following homology directed repair (HDR) have a contrasting highlight. PCR primers set 1 (F1, R1) and set 2 (F2, R2) are indicated as arrows. Also shown is the single-stranded oligonucleotide (ssODN; bottom) with variant sequence (pale nucleotides) used to introduce a specific mutation (*) designed to generate a stop codon and a *Bcl*I restriction site (TGATCA; underlined), while abolishing the native *Pfl*M1 site. **B)** PCR amplicon sequences from SCREAM forward stage with ssODN. Aligned amplicon sequences of clones obtained after electroporation of CRISPR/Cas9 RNPs in a 50 *μ*L reaction (10^8^ cells) coupled with the NIA1-STOP-BclI ssODN, which targets the *NIA1* gRNA target region, and plating on five chlorate selection plates. Note that of the mutants with a *Bcl*I site, 6/7 display only the expected AT.A change. Genome changes are in green, gRNA in blue, protospacer adjacent motif (PAM) in magenta. **C)** SCREAM reverse stage selection plates (digitally extracted for comparison). Nitrate-utilizing (*NIA1*^+^) clones are selected on nitrate selection plates following electroporation of both a gRNA specific for the *NIA1*^−^ mutant sequence, as well as the wild-type sequence ssODN which was used to revert the NIA1_STOP_Bcl1 clone to a wild-type *NIA1*^+^ phenotype. **D)** PCR amplicon sequences of clones obtained from ssODN-mediated reversion to the wild-type phenotype. Note that 10/11 mutants (i.e. excluding Clone No. 3) have been edited back to the original wt GGACG sequence (light blue).

Having established that the chosen gRNA could successfully target *NIA1*, we next used ssODNs (Fig. 1B) to produce a specific targeted alteration to the *NIA1* gene. At the protospacer adjacent motif (PAM) site a TGG→TGA stop codon replacement eliminates the native *Pfl*MI restriction site and terminates translation (Fig. 2A). Two additional base pairs were changed so that a *Bcl*I site was created (Fig. 1B). Consequently, in candidate mutants the *NIA1* PCR amplicon (primers: Table 2) could be assayed by the presence of a *Bcl*I site, and/or loss of the *Pfl*MI site. Amplicon sequencing was therefore necessary only for candidates which were likely to be correct, making the process fast and efficient. Most importantly, the completely defined nature of the mutation meant that it could be precisely targeted by a standard “reverse gRNA” and ssODN to revert the sequence to the wild-type.

**Table 2.** PCR primers used for amplification of edited regions

Primer Name	Amplicon Length*	Sequence (5′-3′)
SeqF NIA1A	704	ACTTTGACCATGGACGAG
SeqR NIA1A		GTCAGAGAGGTGCCGTAC
SeqF NIA1B	547	CAACAAGCCGTTGACTTTGA
SeqR NIA1B		GGCATACATGCACTCACACC
LHCBM1F	1426	GCTGCGCATAGATTTTCTCC
LHCBM1R		GCTACTGACGCAACCAGACA
LHCBM2F	1188	GTGACTTTGAACCGCTGTGT
LHCBM2R		GTGGATCAGGTTCTCGTTGC
LHCBM3F	927	TGGCAGTGTTCTAGTCAGCA
LHCBM3R		AAGA TCA TGCACCCTTCCCA
STM6F	344	CAGTTGTATCGCCAAAGTATCCAC
STM6R		GGATACGCTAGCAGAACCTGTC
APRT-F	920	ATGGCTGACGTTGAGGC
APRT-R		TGCTGCCGTCTTCACAAA
LHCA2-F	514	GAGCTTTGTCCGGAGTTACG
LHCA2-R		AAAATGCAAAACCAGCCTGT
LHCA9-F	711	TTAACCGGCCAAGAAAACAG
LHCA9-R		TCTTCTCGGCCTCAGCATA

The control (“no RNP”) plates showed ∼50 chlorate resistant colonies per plate (∼2.5 per million cells transfected). In contrast, colonies on plates transfected with RNPs and ssODN were too dense to be individually isolated. Therefore, an aliquot of cells was taken from each plate and diluted until single colonies could be identified. In this case, colonies from different plates likely represent independent clones. However, given the large number of colonies observed, even colonies from densely grown secondary plates are probably independent. All chosen colonies (103 across the 5 plates) grew on TAP (Tris-Acetate-Phosphate; see Material and methods), but no colonies grew on nitrate, indicating that most colonies were CRISPR-induced, rather than spontaneous mutations (many of which can still grow on nitrate despite being chlorate-resistant; Supplementary Appendix S2). To confirm this, genomic DNA was extracted, and amplicons from eight mutants were sequenced (Fig. 2B). One clone had a wild-type *NIA1* sequence suggesting that it was a spontaneous mutant. All other clones contained the ssODN sequence at the gRNA target site (including the *Bcl*I restriction enzyme site) leading to premature truncation of nitrate reductase. Of these 7 ssODN modified clones, two had additional mutations. One had a GCG > CCC mutation which would not prevent reversion via a wild-type ssODN, while the other had an insertion difficult to revert to the wild-type sequence. Consequently 6/8 usable mutants were obtained for this forward SCREAM step. A clone with the desired ssODN correctly incorporated (ΔN21) was chosen and designated the “*NIA1*-STOP-*Bcl*I” mutant for future experiments.

### Reversion of mutated sequence to wild-type: reverse SCREAM stage

The *NIA1*-STOP-*Bcl*I clone chosen above was used to test the reverse SCREAM stage, that is, reversion to wild-type, via a ssODN that restores the WT sequence (Table 3; Fig. 1B (lower panel), Fig. 2C) and a specific mutant-targeted gRNA (NIA1_Revert gRNA, Table 1) targeted to the newly introduced target region of the chosen *NIA1*-STOP-*Bcl*I clone (Table 1). The ssODN included a silent C > G transversion on the 5′ arm (see Fig. 1) and a silent G > C on the 3′ arm, solely to test for insertion of the ssODN sequence.

**Table 3.** Single-stranded oligonucleotides (ssODN) used to direct CRISPR/Cas9 gene editing

Gene	ssODN Name	ssODN purpose	Sequence 5′-3′
*NIA1*	NIA1_STOP_BclI	Insert STOP codon & Insert *Bcl*I site	CTGAAGAAGAGCATTGGCTTCAACTGGGGCCCTTGTGCCACCAGCACCACCTACTG**AT**C**A**GGCGTGCGGCTGCGCGACCTGTTGCAGCACGCCG
*NIA1*	NIA1_Revert-to-WT	Target mutant, and revert to WT	CTGAAGAAGAGCATTGGCTTCAACTGGGGCCCTTGTGCCAC**G**AGCACCACCTACTG**GA**C**G**GGCGTGCGGCT**C**CGCGACCT
*APRT*	APRT_STOP_BclI	Insert STOP codon & insert *Bcl*I site	G*G*GTATTCTGTTCTGGGATGTCACCACCATCATGCTGAACCACCAG**TGAT**CAGTACAGCATTGACCTGTTCGCTGAGCAGTACAAGGACAAGAA*G*A

Bold and underline indicates base pair changes that are introduced by the oligonucleotide.

Asterisk (*) indicates base pairs that were synthesized with phosphorothiolate links.

The *NIA1*-STOP-*Bcl*I cells were grown in Nitrate Medium (NM; TAP containing 5 mm KNO_3_ instead of NH_4_Cl) with 2 mm urea as the nitrogen source for the *NIA1*^−^ mutants. Electroporation was conducted in NM + urea medium with 40 mm sucrose added. Although nitrate cannot be used by the *NIA1^−^* mutants, it upregulates the *NIA1* locus via the transcriptional regulator *Nit2* (Fernández and Matagne 1986; Galvan et al. 1992), whereas ammonium ions (e.g. in TAP) would repress the *NIA1* locus (Sanz-Luque et al. 2013). An open locus is desirable for effective editing by Cas9 (Handelmann et al. 2023).

Following overnight incubation, the cells were replated on six Nitrate Medium plates containing only 5 mm KNO_3_ (i.e. no urea) to select for cells able to utilize nitrate (i.e. with an intact *NIA1* gene). As expected, no colonies occurred on the control plates, but the RNP plates contained many (Fig. 2C), suggesting that this selection protocol is very clean. Eleven colonies were checked by PCR followed by *Bcl*I digestion; all were *Bcl*I-resistant signifying alteration of the target locus from *NIA1*^−^ to *NIA1*^+^.

Sequencing revealed that 10/11 mutants contained the reverted *NIA1* WT sequence (Fig. 2D), for a success rate ∼90%. Clone 3 (Fig. 2D) retained the TGA stop codon and was thus out of frame. It is not known such a clone survived; potentially it has a restored *NIA1* gene but possesses a mutated PCR primer site, failing to amplify efficiently. The observed amplicon may then have been derived instead from underlying wild-type cells which temporarily survived on organic nitrogen from nearby dead cells. Of the 10 successfully reverted clones, 5 contained the upstream C > G silent base pair and one contained the 3′ G > C silent base pair change that confirmed the use of the ssODN as a template. The absence of clones containing both changes suggests that typically, ssODNs are partially digested during template guided repair. In summary, the forward (chlorate selection) step successfully resulted in ssODN incorporation and *NIA1* inactivation (∼60% success rate for the PCR-screened and selected clones), whereas the subsequent reverse (nitrate selection) step successfully resulted in the genome sequence reverted to wild-type, with restoration of the *NIA1* gene (90% success rate).

### A complete SCREAM cycle with sequential modification of two target genes

Having established that our chosen gRNAs and ssODNs were able to drive a full SCREAM cycle with the *NIA1* gene, we next employed the cycle to co-select for CRISPR-induced mutations in specific *Chlamydomonas* genes for which mutant selection was otherwise difficult or impossible.

As part of another study into hydrogen production in algae, we required the production of a sequential modification of the genes encoding *LHCBM2* (*light harvesting protein B M2*) and *STM6* (also known as MOC1; *mTERF-like protein of Chlamydomonas-1*). The *LHCBM2* gene encodes the major light harvesting complex II protein 2 of *Chlamydomonas*, one of nine homologous major light harvesting genes of PSII (Sheng et al. 2019). Because of redundancy with eight other homologous LHCII genes, and an unknown phenotype, a screening assay for *LHCBM2* mutants was problematic. The *LHCBM2* mutant was also unavailable in the CLiP collection (Li et al. 2019) and was therefore created via CRISPR/Cas9 gene editing.

In contrast, the *STM6* (*MOC1*) gene knockout is the causative mutation in the *stm6* (*state transition mutant 6*) strain (Schönfeld et al. 2004). The *stm6* strain has a deficient mitochondrial alternative oxidase system (Wobbe and Nixon 2013) and produces high levels of hydrogen (Kruse et al. 2005). We employed SCREAM to combine both mutations in a common CC-1883 background.

#### Target gene 1: LHCBM2

**Figure 3.**
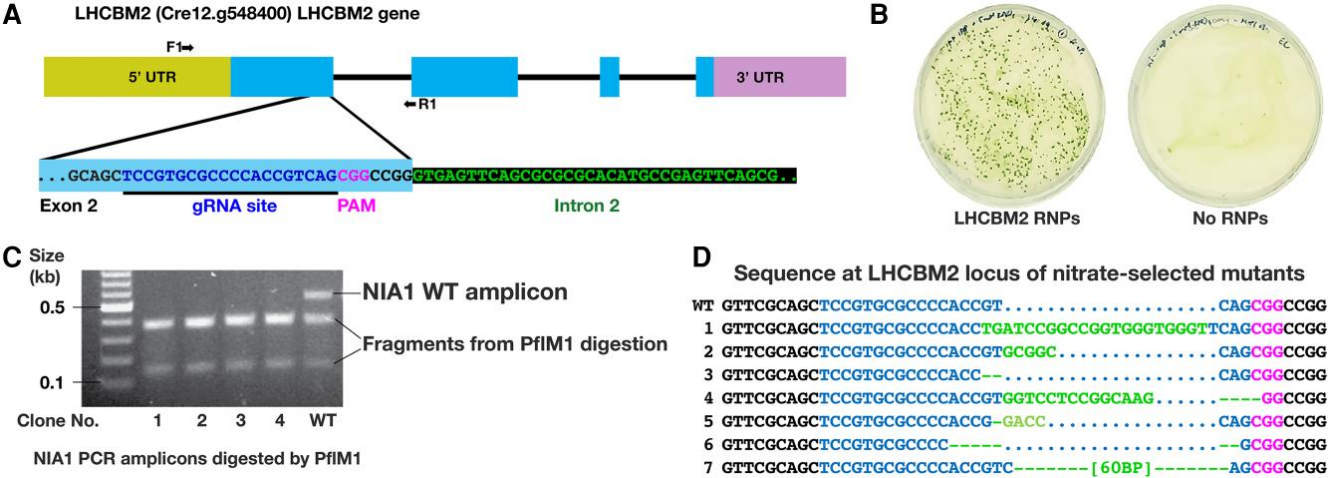
Use of SCREAM to target a nonselectable gene (*LHCBM2*). **A)** Diagram of the *LHCBM2* gene structure (top; exons as blocks, narrow line for intronic regions) and the DNA sequence within the expanded Exon 1:Intron 1 region (bottom) including the guide RNA (gRNA) target site (underlined), and the protospacer adjacent motif (PAM). PCR primers (F1, R1) are indicated with arrows. **B)** Transfection plates selected on nitrate for *NIA1* Reversion/*LHCBM2* knockout via SCREAM (plate images digitally extracted for comparison). Electroporation employed 100 *µ*L of cell suspension (27.5 ×10^6^ cells per reaction), 50 *µ*L ribonucleoproteins (RNPs), and 5 nmol of LHCBM2 gRNA (Table 1). **C)** Clones tested for *Pfl*MI digested PCR amplicons showing restoration of the wild-type *Pfl*MI site. Although not employed here, the 5′ transversion in the *NIA1* gene also created a new restriction enzyme site (*BauI*), which offers an additional restriction enzyme based check for the presence of this sequence in the PCR amplicon. **D)** Sequence of wild-type vs Δ*LHCBM2* knockout clones at the target site showing target gene indel formation. Genome changes are listed in pale green, gRNA target sequence in blue, PAM in magenta.

For the first target gene knockout (KO), we transfected an anti-*LHCBM2* gRNA (Table 1; Fig. 3A) into the *NIA1*-disabled mutant created with the NIA1_STOP_BclI ssODN, so that the reversion-to-wildtype “reverse” SCREAM stage could be used for the first target gene modification. RNPs for the *LHCBM2* and *NIA1* target regions were assembled separately with Cas9, and then combined with the *NIA1*_Revert-to-WT ssODN (5 nmol). These were transfected as before, and selected on nitrate-containing agar. No colonies were obtained when the RNP mix was omitted, whereas 3,380 colonies were obtained on the two RNP plates (Fig. 3B), which represented around 0.56% of the entire population of viable cells. Twenty-two colonies were selected for PCR analysis. All amplicons were successfully cleaved by *Pfl*MI (Fig. 3C), suggesting restoration of the original *NIA1* wild-type sequence by ssODN-mediated repair. Sanger sequencing (Fig. 3D) of the *LHCBM2* PCR amplicons of these clones revealed that 7 clones also contained edited *LHCBM2* target genes, yielding a target co-editing efficiency ∼32%.

Figure 3D shows that the mutated *LHCBM2* genes had indels at 1 to 5 nucleotides from the 5′ end of the PAM site. In one case (clone 7), part of the ssODN sequence used to revert the *NIA1* sequence to the WT was inserted at the Cas9 cleavage site of the *LHCBM2* gene, illustrating a NHEJ/TMEJ-mediated knock-in effect. Of the mutants with successful indel formation at *LHCBM2*, all had the *NIA1* sequence returned to the wild-type sequence, with 5/7 containing the silent 5′ check nucleotide and 2/7 the 3′ check nucleotide, confirming that the supplied ssODN was used as the repair template. Although the *LHCBM2* KO was produced using a single gRNA, we routinely now employ two gRNAs per target gene for knockouts, since the resulting large change in PCR amplicon size is readily apparent, simplifying screening.

#### Target gene 2: STM6

**Figure 4.**
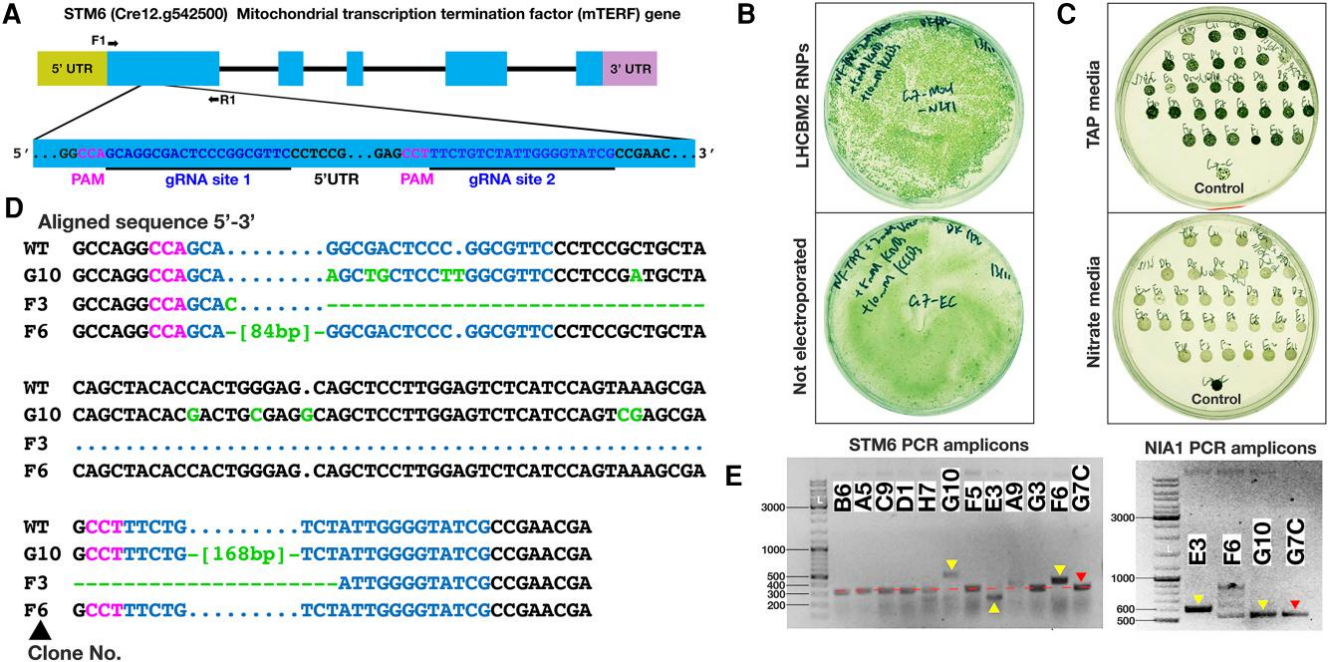
Use of SCREAM with dual guide RNAs (gRNAs) to sequentially target the *STM6* gene in the *LHCBM2* mutant (*LHCBM2*/*STM6*). **A)** Diagram of the *STM6* gene structure (top; exons as blocks, narrow line for intronic regions) and the DNA sequence within the expanded Exon 1 target regions (bottom) including the two gRNA target sequences (underlined), and the protospacer adjacent motif (PAM). PCR primers (F1, R1) are indicated with arrows. The use of two gRNAs for the STM6 exon 1 was designed to enable deletion of the chosen region just downstream of the start codon. Although Cas9 cleavage typically introduces indels, these are usually small in size and run similarly to the unmodified gene on electrophoresis gels. In contrast, Cas9 cleavage at both sites produces a readily detectable change in the PCR amplicon size which is convenient for screening. **B)** Chlorate selection plates showing numerous candidate *NIA1*/*STM6* mutation colonies via SCREAM vs control plates with a general background of dying cells and few colonies (plate images are digitally extracted for comparison in panels B and C). **C)** Clones tested for growth on TAP vs nitrate plates showing loss of ability to grow on nitrate as a sole nitrogen source compared to control (wild-type) spot. **D)** Sequence of Δ*LHCBM2* Δ*STM6* clones; Genome changes are listed in pale green, gRNA in blue, PAM in red. **E)** Analysis of candidate *STM6* disruption mutant clones by PCR. Red arrows indicate amplicons from the starting cell line (parent Δ*LHCBM2* clone G7C), text in white boxes indicates clone numbers. In the left panel, yellow arrows show altered *STM6* PCR amplicon size, signifying disruption of the locus. In the right panel, yellow arrows highlight *NIA1* amplicons, with indels apparent in clones E3 and F6 but not in G10.

In the previous step, a CC-1883 *NIA1*^−^ mutant was engineered using SCREAM to simultaneously restore the *NIA1^+^* genotype and to knockout the *LHCBM2* gene. To further engineer a *STM6* (Δ*STM6*) knockout, a clone (G7) of the *LHCBM2* knockout cell line was then modified via forward SCREAM (*NIA1^+^*→*NIA1^−^*), using chlorate selection, to create the triple mutant CC-1883 *NIA1^−^ LHCBM2^−^ STM6 ^−^* for a complete SCREAM cycle. Figure 4A shows the two *STM6* gRNAs employed against *STM6* exon 1, along with the *NIA1* gRNAs (Table 1) and the NIA1_STOP_BclI ssODN (Table 3). Deletions between these gRNA sites produce a large readily detected change in the amplicon size. As shown in Fig. 4B for one plate (of 4), many thousands of colonies resulted (estimated >5,000 in total), far in excess of the chlorate spontaneous mutant background (∼20 colonies per million cells plated).

Four 96-well plates of colonies were picked (one from each of the four selection plates) into TAP medium, each containing 95 colony wells and one control (ΔLHCBM2 clone_G7) well. Colonies were transferred to agar plates containing nitrogen free TAP supplemented with 5 mm KNO_3_, to identify colonies disrupted in nitrate assimilation. As shown for one set of clones (Fig. 4C) no colonies grew effectively on nitrate, compared to the G7 (ΔLHCBM2 clone; see Fig. 3D) control. This suggests that the number of spontaneous chlorate mutants on the +RNP plates was very low and can be effectively disregarded. Around 300/396 colonies showed unambiguous disparity between TAP and nitrate plates and were maintained, in case the mutation frequency of the target gene was low. Twelve randomly selected clones were checked by PCR amplification of the *STM6* target locus (Fig. 4E). Of these, 3/12 (i.e. ∼25%) showed large changes in the *STM6* PCR amplicon size, suggesting that either a large insertion occurred (amplicons larger) or that both gRNAs had caused double strand breaks in the genome (amplicons smaller), which had been repaired by NHEJ/TMEJ. Sanger sequencing of these three clones revealed that clones G10 and F6 contained insertions directly bridging the two gRNA sites, while E3 contained the desired deletion between the two gRNA sites (Fig. 4D). This supports the view that the altered PCR amplicon sizes were due to dual gRNA targeting events. Of the clones that did not show large changes in amplicon size, some will likely contain indels that also inactivate the target gene, although these were not sequenced. It should also be possible to create precise deletions by using bridging ssODNs. The G10 and E3 mutations terminate in exon 1, while F6 has a 28 amino acid insertion in exon 1.

These three clones were also analysed for changes at the *NIA1* locus. Clone G10 had a wild-type *NIA1* PCR amplicon size. Chlorate resistant was explained by both point mutations and a single G insertion 1 nt before the PAM site which created a frameshift. Clones E3 and F6 contained insertions, visible on PCR, which disrupted the *NIA1* locus.

### A SCREAM cycle with sequential modification of three target genes

**Figure 5.**
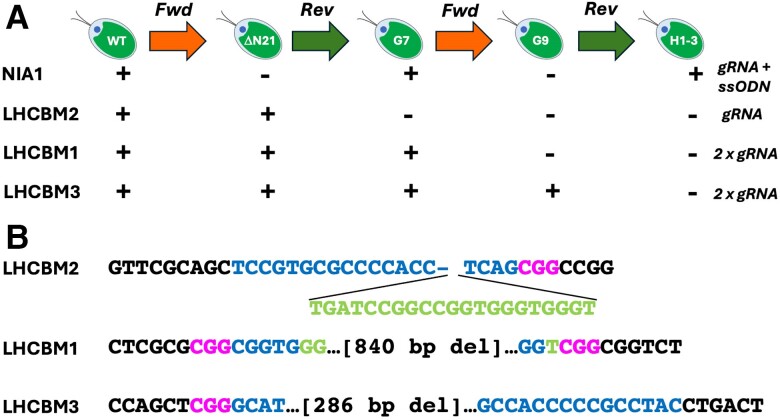
Sequential deletion of *LHCBM1* and *LHCBM3* on the *LHCBM2* knockout background, with restoration of wild-type *NIA1*. **A)** Summary diagram of the strategy used to convert the wild-type CC-1883 first to a precise (single-stranded oligonucleotide-mediated) *NIA1* mutant (clone “ΔN21”), then to the *LHCBM2* knockout (clone “G7”; numbered as clone 1 in Fig. 3D) as described in Fig. 2, followed by further rounds of SCREAM to delete the *LHCBM1* (clone “G9”) and then the *LHCBM3* gene (clone “H1-3”) with restoration of the wild-type *NIA1* gene. “Fwd” and “Rev” denote forward and reverse SCREAM stages, respectively. **B)** Final sequence at the *LHCBM2, LHCBM1* and *LHCBM3* loci in clone H1-3. Genome changes in green, guide RNA (gRNA) sequences in blue, protospacer adjacent motif (PAM) in magenta. The insert in the *LHCBM2* knockout clone G7 is identical in sequence to a small intergenic region on *C. reinhardtii* chromosome 12 between Cre12.g490500 and Cre12.g490450. The deletions in *LHCBM1* and *LHCBM3* were each generated using two gRNAs (listed in Table 1).

The *LHCBM2* gene knockout described above was also employed as a basis for creating further light harvesting gene knockouts using SCREAM (Fig. 5A). Since the *LHCBM2* KO was created from a *NIA1* knockout, it has a wild-type *NIA1* gene. Therefore, the NIA1_Disrupt gRNA and corresponding ssODN were used in conjunction with two gRNAs directed against exons 1 and 3 of the *LHCBM1* gene to create deletions of exons 1 to 3. Following chlorate selection, 12,640 colonies were obtained (compared to 264 and 565 on control plates) for a survival rate of ∼0.37% of the cells plated and a spontaneous mutation background of 2% to 3%. PCR analysis was conducted on 69 randomly selected colonies, of which 32 (46%) showed deletion of the region between the two gRNAs in the *LHCBM1* gene, resulting in a truncated LHCBM1 protein. Again, PCR amplicons not showing a deletion were not sequenced so that small indels would not have been detected. The co-targeting rate is therefore conservative. Of these, *NIA1* PCR amplification and testing with B*cl*I/P*fl*MI digestion led to selection of 4 clones with the correct screening characteristics. Of these, 3 clones had the *NIA1*_STOP_BclI ssODN correctly incorporated and one had additional point mutations.

For the third knockout, a double knockout clone (*LHCBM2/LHCBM1* clone G9-3) was chosen which has the *NIA1*^−^ phenotype conferred by the NIA1_STOP_BclI ssODN, and an 840 bp deletion in the LHCBM1 gene. The NIA1_Revert-to-WT ssODN and gRNAs were used in conjunction with two gRNAs directed at the *LHCBM3* gene with selection on nitrate plates for reversion to the wild-type NIA1 gene. Nitrate plates showed 0 colonies on the control plates and 1,153 colonies on plates with RNPs, with a survival rate for cells plated of 0.13%. Of 48 randomly picked colonies, 16 showed evidence of *LHCBM3* deletion (5 deletions and one insertion) and following PCR screening, 6 clones were isolated with both correct restoration of the wild-type *NIA1* locus and deletion of *LHCBM1, LHCBM2* and *LHCBM3* genes. To date, the *LHCBM1* knockout has been described (Elrad et al. 2002) and a representative clone of this triple knockout (clone H1-3; Fig. 5B) is under study in our laboratory. Numbers of successful clones obtained from this experiment and data on the likelihood of ssODN incorporation are provided in Table 4. This demonstrates that SCREAM has the potential to generate indefinite sequences of CRISPR-mediated gene knockouts.

**Table 4.** Summary of mutation rates in SCREAM experiments

Target gene	Type of experiment^a^	Colonies examined	Target gene mutation identified by PCR and/or enzymatic digestion	Target gene mutation (identified by sequencing)^b^	ssODN precisely incorporated in target gene(s)
*NIA1*	*NIA1* KO	48	23	23/23 sequenced^c^	N/A
*NIA1*	RTW	24	11	11/11 sequenced	10/11
*LHCBM2*	RTW	22	N/A	7/22 sequenced	22/22
*LHCBM 3*	*NIA1* KO	100	18	18/18 sequenced	1/18
*LHCBM 1*	*NIA1* KO	69	32/69^c^	32/32 sequenced	3/32
*LHCBM 3*	RTW	110	18/110^c^	16/18 sequenced	16/16
*LHCBMs 1 & 3*	RTW multiplex	94	39/94	1 gene disrupted: 39 2 genes disrupted: 4	2/4 double mutants
*STM6*	*NIA1* KO	11	3	3/3 sequenced	0/3
*APRT* [NM]	*NIA1* KO	25	9/25	8/12 sequenced 11/25 including PCR	2/25 (*APRT*) 2/25 (*NIA1*)^d^
*APRT* [TAP]	*NIA1* KO	25	4/25	7/24 sequenced 8/25 including PCR	0/25 (*APRT*) 5/25 (*NIA1*)^d^

^a^“*NIA1* KO” = Cas9 knockout of *NIA1* with selection on chlorate media. “RTW” = Reversion to wild-type by restoring *NIA1* wt sequence and selecting on nitrate media. ^b^By change in amplicon size—small indels could have been present in other colonies but would not have been detected. ^c^Clones were screened by PCR and digestion of the target amplicon. Only clones meeting these criteria were sequenced. It is unknown how many unsequenced clones also harboured mutations. ^d^Correct HDR-mediated NIA1_STOP_BclI ssODN incorporation in the 25 clones examined.

### Reaction optimization

As our standard CRISPR reaction generated far more *NIA1*-engineered clones than required to isolate a mutated target gene, the reaction was scaled down, to 5 million cells and one tenth as much RNP mix (see Material and methods). A voltage titration was conducted (Supplementary Fig. S3A), estimating the actual yield of presumed CRISPR-edited *NIA1^−^* colonies, rather than employing plasmid-based transfection (Supplementary Fig. S2). Electroporation of 25 *µ*L of cell-RNP mixture in a 1 mm cuvette still yielded over 20,000 colonies (∼0.4% of cells electroporated) and was more economical. With electroporation, efficiency increases with voltage, but this is offset by increased cell killing at higher voltages (Supplementary Fig. S3B). The most efficient conditions represent a compromise between these two variables. Here, CC-1883 yielded most colonies at 200 V 25 *µ*F, infinite resistance, likely a reasonable setting for most *cw15* strains. However each strain (especially those with intact cell walls where efficiencies will be low; Yamano and Fukuzawa (2020)) will require electroporation calibration.

### Frequency of co-editing of target genes

An important variable in SCREAM is the frequency of target gene co-editing compared with the *NIA1* marker, since editing frequency is dependent both on the target gRNA and the accessibility of the target site (Handelmann et al. 2023). The adenine phosphoribosyltransferase (*APRT*) locus was chosen as an independently selectable target so that frequency estimates could be performed simply using colony counting. *Chlamydomonas* cells with *APRT* knockout mutations can be selected on the toxic purine analogue 2-fluoroadenine (2FA) since wild-type cells take up 2FA and incorporate it into their genomes, whereas mutants rely exclusively on de novo adenine synthesis (Littlefield 1966). *APRT* had been originally considered as an alternative selection paradigm for SCREAM experiments but the selection of *APRT*^+^ cells may require treatment with both azaserine and alanosine to block the de novo pathway and simultaneous supplementation with adenine, which is a more complex system than the *NIA1* system. It is, however, a potential selective marker alternative to *NIA1* in the case of cell lines lacking a functional nitrate assimilation pathway. The *APRT* locus has already been successfully used in CRISPR experiments in *Chlamydomonas* (Guzmán-Zapata et al. 2019) and *Chlorella* (Kim et al. 2021).

According to Ferenczi et al. (2017) inclusion of a single-stranded oligonucleotide (ssODN) for homology-directed repair increases the editing frequency of the target gene over use of a gRNA alone. We therefore compared RNP electroporations with and without *APRT*-directed ssODN, again using colony counting on selective media to score likely candidates, with the aim of improving the ratio of target (*APRT*) to *NIA1* mutants to improve screening efficiency.

**Figure 6.**
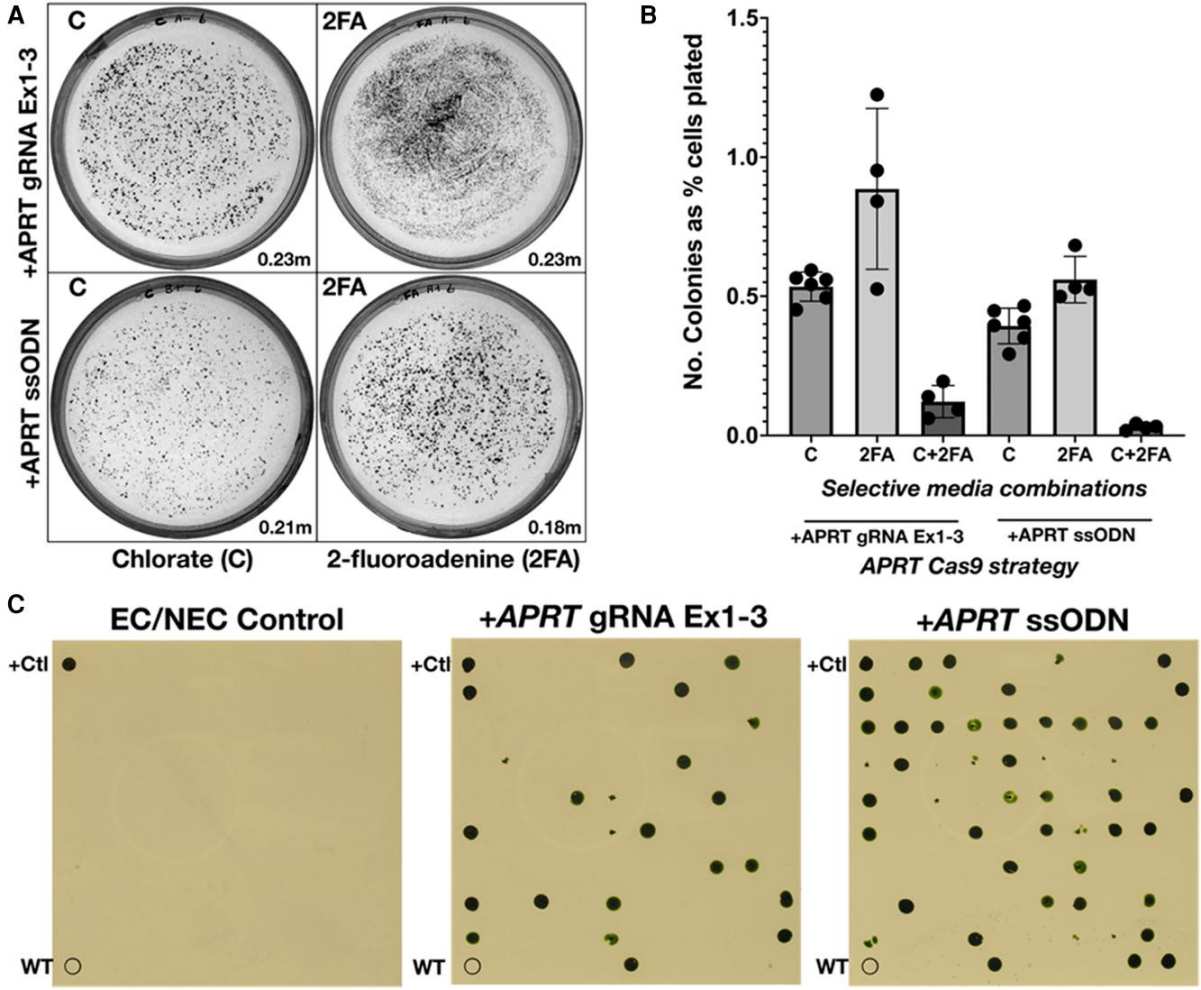
Effect of single-stranded oligonucleotides (ssODN) on target gene modification efficiency. **A)** Representative selection plates showing RNP electroporation with either two *APRT*-directed gRNAs (against exon 1 and exon 3; “gRNA Ex1-3”) to produce gene deletions, or with one gRNA and an *APRT*-directed ssODN to insert a stop codon (“ssODN”). All experiments included *NIA1*-directed ssODN and *NIA1*-directed ribonucleoproteins (RNPs). Selection plates contained chlorate (“C”) to select for *NIA1* disruption or 2-fluoroadenine (“2FA”) to select for *APRT* disruption. Each plate represents ∼4% of each RNP electroporation (∼0.2 million cells; for specific values see bottom right-hand corner of each plate panel). Inclusion of the *APRT*-directed oligonucleotide led to a slight decrease in the total number of mutants obtained compared with dual guide RNAs (gRNAs). Plate images were digitally extracted for comparison and images were processed as described in the Materials and methods. **B)** Colony counting of the full dilution plate series was conducted to estimate the frequency of candidate *NIA1* edited clones (“C”: resistant to chlorate), candidate *APRT* edited clones (“2FA”: resistant to 2-fluoroadenine), and candidate *NIA1 + APRT* doubly edited clones (“C + 2FA”: resistant to both chlorate and 2-fluoroadenine). Clone numbers were estimated from selection plates using ImageJ. Circles represent data from independent plates (error bars show ± 1 SD from mean). **C)** Randomly selected chlorate resistant colonies were picked, grown in TAP and gridded out on 2-fluoroadenine containing selective media. To validate the colonies on the grid, each plate contained a wild-type CC-1883 negative control (“WT”) and a previously isolated 2FA-resistant positive control (“+Ctl”). Randomly picked colonies consisted of (i) chlorate-resistant colonies taken from control plates spread with nonelectroporated (“NEC”) cells or the non-RNP containing electroporated (“EC”) cells; (ii) the RNP electroporations with only *APRT*-directed gRNAs (“+*APRT* gRNA Ex1-3”) or (iii) RNP electroporations containing 0.5 nmol *APRT*-directed ssODN (“+*APRT* ssODN”).

Cells were plated on selective media comprising chlorate, 2-fluoroadenine or both, using a dilution series to generate plates with a suitable density for accurate colony counting, to estimate the mutational frequency of *NIA1* and *APRT* from the same electroporation reaction (Fig. 6). Colony counts from control reactions lacking RNPs were used to measure spontaneous mutation rates for each condition but showed negligible numbers of colonies on single selection plates (<0.0005% of cells plated) and no colonies on double selection (2-fluoroadenine + chlorate) plates. The spontaneous mutant background can be therefore be disregarded.

On the most densely populated chlorate selection plates, ∼5× as many colonies occurred as on the corresponding double selection plates, suggesting ∼15% to 20% of cells were edited in both the *NIA1* and *APRT* loci (Fig. 6, A and B). However, on the more sparsely spread double selection plates (where colony quantification is most accurate) the colony numbers, as a percentage of cells spread, were dramatically reduced. This suggests that single CC-1883 cells sparsely plated on selective media cope poorly with the simultaneous presence of both selection agents, even when genuinely edited at both loci. The true rate of double locus editing was thus presumed to be higher than indicated by the double selection plates. To improve quantification accuracy, 95 individual colonies were picked from single selection plates and gridded out on the opposite selection media, where the appearance of colonies identified clones that had both loci edited (Fig. 6C).

As expected, none of the 95 chlorate resistant colonies isolated from control plates were resistant to 2-fluoroadenine (Fig. 6C left hand panel). In contrast, colonies derived from the CRISPR electroporation plates showed significant levels of cross-resistance (Fig. 6C middle and right hand panels). From electroporation reactions employing dual gRNAs instead of the *APRT*-directed ssODN, 16 out of 95 picked chlorate-resistant colonies were also strongly resistant to 2-fluoroadenine. When a single gRNA and the *APRT*-directed ssODN was included in the electroporation, 39 out of 95 picked chlorate-resistant clones (40.6%) were simultaneously resistant to 2-fluoroadenine, demonstrating a 2.5-fold improvement in the efficiency of co-editing the *NIA1* and *APRT* loci.

Inclusion of the *APRT*-targeting ssODN led to a higher frequency of 2-fluoroadenine-resistant clones following chlorate selection, even though the absolute number of colonies obtained was somewhat lower (∼0.7× Fig. 6B) when the ssODN was included. However, we note that this result depends on the relative activity of the ssODN and the dual gRNAs for the *APRT* gene, and probably cannot be generalized.

Together with the higher primary colony numbers on single selection plates, this suggests that *APRT* is a more easily mutated locus than *NIA1*, possibly because the metabolic role of APRT ensures that the locus is usually open, whereas *NIA1* is an inducible gene and due to stochastic effects on gene expression, may be closed in a proportion of cells even under inducing conditions (Ross et al. 1994; Finn and Misteli 2019). In the converse experiment (Supplementary Fig. S4), 2 out of 95 picked 2-fluoroadenine colonies were simultaneously resistant to chlorate when the *APRT* ssODN was not included, while inclusion of the ssODN led to 4 of the 95 2-fluoroadenine resistant colonies also displaying chlorate resistance.

### *NIA1* locus editing is increased by gene activation

**Figure 7.**
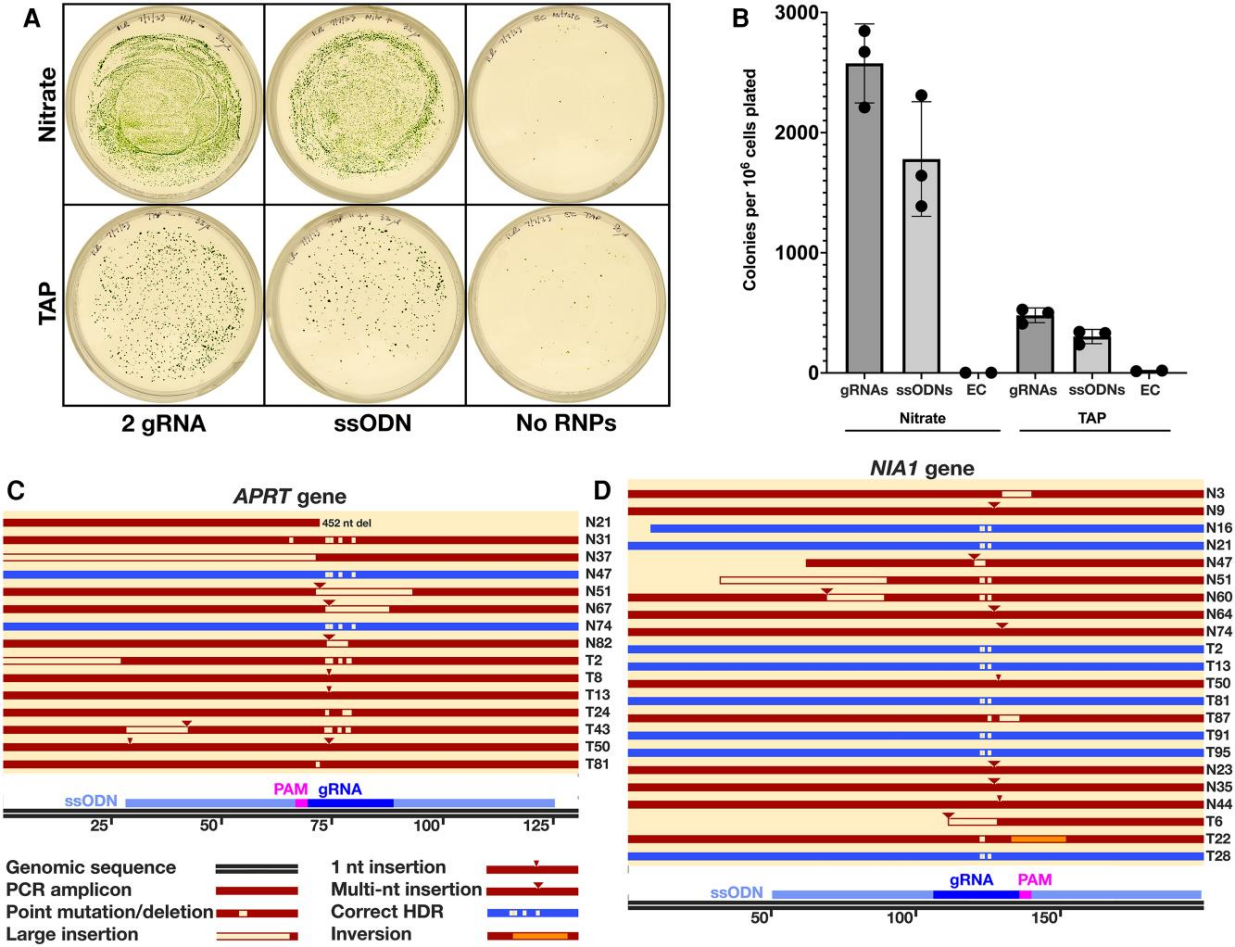
Assessment of candidate APRT knockout (KO) clones generated under *NIA1*-inducing or *NIA1*-repressing conditions. **A)** Representative plates showing chlorate-resistant colonies resulting from pretransfection growth in Nitrate Medium (nitrate reductase induced) vs TAP (nitrate reductase repressed), with either dual guide RNAs (“2 gRNA”) against the target gene or a single stranded oligonucleotide (“ssODN”) and a single gRNA. Ribonucleoprotein (RNP) transfected plates represent one eighth of the electroporated cells. Electroporation controls (EC) represent one quarter of the control cells. **B)** Colony quantification of dilution plates from Nitrate vs TAP experiment. Circles represent data from independent plates (error bars show ± one standard deviation from mean). **C)** Alignment of *APRT* PCR amplicons (horizontal bars) for candidate *APRT* KO clones (clone numbers at RHS). Fifty chlorate resistant candidate *APRT* knockouts were checked by PCR, and 15 amplicons close to the wild-type size were sequenced. Twenty one out of 50 candidates (∼40%) had evidence of *APRT* disruption around the gRNA site. Clones N9, N16, and N60 did not yield a clear *APRT* amplicon, likely due to lost primer sites. PCR evidence of large insertions was obtained for clones T1 and T66. Sequence data were obtained for the remaining 15 clones. In total, 2/21 (blue bars) (∼10% of mutants) had evidence of ssODN-mediated homology directed repair (HDR). **D)** Alignment of *NIA1* PCR amplicons (horizontal bars) for 22 candidate APRT KO clones for which PCR amplicons were close to the WT size (clone numbers at RHS). All *NIA1* amplicons showed *NIA1* disruption around the gRNA site including at least 8/50 (∼16%; blue bars) with evidence of ssODN-directed HDR at the gRNA site. All 50 *NIA1* amplicons showed *NIA1* disruption by either PCR or sequence analysis.

Using chlorate-resistant colonies to estimate gene editing frequency, and again using *APRT* to examine co-editing frequency, we compared the relative efficiency of *NIA1* editing in CC-1883 cells grown in nitrate-containing, ammonium depleted media (in which the *NIA1* locus is known to be induced; reviewed by Sanz-Luque et al. (2015)) vs those grown in TAP, in which the presence of ammonium suppresses the *NIA1* locus (Fig. 7, A and B). For cells grown in nitrate media, ∼5 times as many colonies appeared compared to those grown in TAP (Fig. 7, A and B), supporting the idea that gene activation (and thus chromatin accessibility of the locus) is an important factor in the frequency of gene targeting by CRISPR (for target genes as well as to the *NIA1* marker). This in turn indicates that SCREAM should, if possible, be carried out under conditions in which the target gene is actively being transcribed. Nevertheless, in targets examined to date, we have successfully obtained target gene modification even under conditions where the gene is expected to be repressed. We again noted the effect of providing the *APRT* ssODN on co-disruption of *APRT*, with chlorate resistant clones having ∼41.6% (ssODN) vs 16% (2 × gRNAs) disruption of *APRT* (Supplementary Fig. S5) suggesting that ssODN insertion traps Cas9-generated ds breaks that would otherwise be repaired.

### Analysis of *APRT* mutants

From this last experiment, we analysed 50 clones to identify the types and frequency of *NIA1* and *APRT* disruption, to estimate unscreened mutation rates, including ssODN incorporation by HDR. Analysis was conducted using clones from the ssODN plates. Twenty five chlorate-resistant colonies from the Nitrate Medium transfection (NMT; *NIA1* induced) and 25 from the TAP transfection (TAPT; *NIA1* repressed) were analysed by PCR for clearly apparent indels. Those with amplicons close to the wild-type size were sequenced to identify smaller indels or homology directed repair (Fig. 7, C and D and Supplementary Figs. S6 and S7).

Of the 25 NMT colonies, all had *NIA1* gene disruption (and correct *NIA1* ssODN incorporation in 2/25) and 10/25 had *APRT* gene disruption (correct *APRT* ssODN incorporation in 2/25). Of the 25 TAPT colonies, again, all exhibited *NIA1* disruption (5/25 with correct *NIA1_*STOP_BclI ssODN incorporation) and 8 had *APRT* gene disruption (none with correct *APRT_*STOP_BclI ssODN incorporation). We doubt that there is a significant difference in ssODN incorporation between the NMT and TAPT experiments, as several clones were identified in each case where incorporation of the ssODN had occurred but additional point mutations or indels were also present around the gRNA site. Insertions were mainly composed of the cognate ssODN and/or genomic DNA.

Following tests of 2-fluoroadenine sensitivity in the chlorate resistant (*NIA1*-disrupted) colonies, we found that in the Nitrate Medium Transfection (NMT), 11/13 2-fluoroadenine resistant colonies sequenced had disruption of the *APRT* gene at the gRNA site, while all of 2-fluoroadenine sensitive colonies sequenced had the wild-type genotype. However, we noted that in the TAPT experiment, where many fewer chlorate-resistant colonies were initially obtained, while all 2-fluoroadenine sensitive colonies had a wild-type target locus, ∼30% of the colonies that were 2-fluoroadenine resistant lacked *APRT* disruption at the target locus, suggesting that they had acquired mutations elsewhere in *APRT* or in other genes conferring 2-fluoroadenine resistance. This may be related to the fact that the spontaneous rate of 2-fluoroadenine resistance is relatively high (∼1 in 20,000 cells in our experience) and thus more prominent when CRISPR *NIA1* efficiency drops, as in the TAPT (*NIA1*-repressed) experiment.

The high number of *APRT*-disrupted clones displaying partial incorporation of the APRT_STOP_BclI ssODN again supports the view that the higher frequency of ssODN-mediated disruption (compared to using dual gRNAs and no ssODN) observed in Fig. 6, Supplementary Fig. S5 and Fig. 7 is due to unsuccessful HDR-mediated ssODN incorporation into Cas9-mediated ds breaks that would otherwise have been seamlessly repaired by NHEJ/TMEJ, and supports the model of Ferenczi et al. (2021) for ssODN incorporation via the end-joining DNA repair machinery. Notably, inclusion of nonhomologous (i.e. *NIA1*) ssODNs at the *APRT* locus was rare. In general, we prefer the use of dual gRNA approach (which produce large deletions) for permanent gene knockouts even though the frequency is lower, unless the option of reversion of the target gene is specifically desired.

In summary, these results suggest that using the SCREAM technique, electroporation of 5 million cells gives rise to around 25,000 *NIA1* candidate colonies, of which 10% to 40% (depending on the approach) have the *APRT* target gene disrupted. Even allowing for undesirable disruptions (e.g. very large indels, badly placed indels at each locus, short indels that leave the reading frame intact) most of these will be effective knockouts, so that multiplexing (disruption of multiple target genes and/or library construction) is feasible as long as suitably high throughput screening for the target gene is available. In a single CRISPR ssODN-directed experiment, PCR screening of between 10 and 20 chlorate-resistant colonies should suffice to identify a clone with a disrupted *APRT* target gene, while a single 96-well plate should be sufficient to obtain ssODN incorporation at both loci. Broader screening will be required for targets with less accessible loci. We believe that SCREAM efficiency can be further enhanced, and potential directions are discussed in Supplementary Appendix S3.

### Off-target mutation

As homology-directed repair (HDR) is a less frequent outcome than indel formation at Cas9 loci, we often see incomplete or incorrect insertion of ssODN at the cognate target locus. To date, however, we have only twice observed insertion of the *NIA1* ssODN at Cas9-created double-strand breaks in co-targeted genes (or vice versa), which leads us to believe that off-target effects are rare (for further detail, see Supplementary Appendix S4). We attribute this rarity to gRNA design (via Cas-OFFinder; http://www.rgenome.net/cas-offinder/) and consequently, ssODN design, that avoids potential alternate Cas9 target sites. Finally, the use of 3 to 5 independent mutants for subsequent phenotypic analysis should reduce misleading results due to single mutants which contain unanticipated genomic changes. Since traditional transfection experiments clearly show that any supplied DNA has a finite probability of being inserted randomly into the genome (including cellular DNA), only genome sequencing would provide full confidence that unintended mutations are not present in a given clone.

### SCREAM applicability

To date, we have used SCREAM to produce single *Chlamydomonas* lines containing up to five consecutive gene modification events (first, KOs in *NIA1*, *LHCBM1,2,3*, and light harvesting complex A2 (*LHCA2)*; second, KOs in *NIA1*, *LHCBM1,2,3*, and light harvesting complex A9 (*LHCA9))* and we have made at least 14 engineered cell lines (listed in Table 5), which will be described in subsequent publications. We have trialled multiplexing SCREAM (i.e. the simultaneous use of gRNAs against more than one target). For example, *LHCBM1* and *LHCBM3* were targeted in a single electroporation, resulting in both single and double knockouts (Table 4). However, unless target gene conversion efficiencies are very high, the number of PCRs that need to be conducted to find the modified clones rapidly increases with the number of the genes targeted in a single mixed gRNA electroporation (see Supplementary Appendix S3 for further discussion). The simplicity of the SCREAM electroporation and plating process generally makes it easier to modify target genes in sequence, each requiring only a simple screening process, unless a library is specifically desired or a target gene-based screen (e.g. flow cytometry) is available. In contrast, multiple SCREAM experiments targeting a single gene each can easily be run in parallel.

**Table 5.** Summary of knock-out lines created using SCREAM to date

No.	Initial strain	Final genotype	Knockout method
*1*	CC-1883	*NIA1^−^*	**Indel** (*NIA1 Disrupt*) and separately using **ssODN** (gRNA *NIA1 Disrupt* + ssODN *NIA1_STOP_BclI*)
*2*	CC-1883	*APRT^−^*	**Indel** (*gRNAs APRT-Ex1 + APRT-Ex3*) and separately using **ssODN** (gRNA *APRT-Ex1* + ssODN *APRT_STOP_BclI*)
*3*	CC-1883^a^	*NIA1^−^, APRT^−^*	**Indel** (*gRNAs APRT-Ex1 + APRT-Ex3*) and separately using **ssODN** (gRNA *APRT-Ex1* + ssODN *APRT_STOP_BclI*), with NIA1 gRNAs and ssODN^b^
*4*	*NIA1^−^*	*NIA1^+^, LHCBM1^−^*	**Deletion** (gRNAs *L1-Ex1* and *L1-Ex3*)
*5*	*NIA1^−^*	*NIA1^+^, LHCBM2^−^*	**Indel** (gRNA *L2-Ex1*)
*6*	*NIA1^−^*	*NIA1^+^, LHCBM3^−^*	**Deletion** (gRNAs *L3-Ex3* and *L3-Ex4*)
*7*	*NIA1^+^ LHCBM2^−^*	*NIA1^−^, LHCBM1^−^, LHCBM2^−^*	**Deletion** (gRNAs *L1-Ex1* and *L1-Ex3*)
*8*	*NIA1^−^*	*NIA1^+^, LHCBM1^−^, LHCBM3^−^*	**Multiplex deletion** (gRNAs *L1-Ex1* and *L1-Ex3; L3-Ex3* and *L3-Ex4*)
*9*	*NIA1^+^ LHCBM2^−^*	*NIA1^−^, LHCBM2^−^, LHCBM3^−^*	**Deletion** (gRNAs *L3-Ex3* and *L3-Ex4*)
*10*	*NIA1^−^, LHCBM1^−^LHCBM2^−^*	*NIA1^+^, LHCBM1^−^, LHCBM2^−^ LHCBM3^−^*	**Deletion** (gRNAs *L3-Ex3* and *L3-Ex4*)
*11*	*NIA1^+^ LHCBM1^−^LHCBM2^−^LHCBM3^−^*	*NIA1^−^, LHCBM1^−^, LHCBM2^−^ LHCBM3^−^, LHCA2^−^*	**Deletion** (gRNAs *Lhca2-Ex1* and *Lhca2-Ex2*)
*12*	*NIA1^+^ LHCBM1^−^LHCBM2^−^LHCBM3^−^*	*NIA1^−^, LHCBM1^−^, LHCBM2^−^ LHCBM3^−^, LHCA9^−^*	**Deletion** (gRNAs *Lhca9-Ex1* and *Lhca9-Ex2*)
*13*	CC-1883	*NIA1^−^, LHCBM9^−^*	**Deletion** (gRNAs *L9-Ex2* and *L9-Ex3*)
*14*	*NIA1^+^ LHCBM2^−^*	*NIA1^−^, LHCBM2^−^, STM6^−^*	**Deletion** (gRNAs *STM6-Ex1A* and *STM6-Ex1B*)

^a^From Item 3 and onwards, NIA1^−^ cell lines employ precise ssODN-mediate STOP codon insertion using the gRNA NIA1_Disrupt and the ssODN NIA1_STOP_BclI, while the NIA1^+^ cell lines have a reversion to wild-type sequence which employ the gRNA NIA1_Revert and the ssODN NIA1_Revert_to_WT. The use of these NIA1 gRNAs and ssODNs is implied but not stated explicitly. The gRNA names are detailed in Table 1. ^b^These ssODNs have 2 phosphorothioate linkages at each end.

Specific gRNAs against the target gene optimized using the many bioinformatic design tools available should generally suffice, especially if more than one gRNA is used. In the case of *NIA1* gRNAs, the relevant gRNAs for the forward and reverse stages are necessarily matched pairs, so optimal design for one gRNA may, in theory, result in less than optimal design of the other pair member. However, higher RNP activity against the *NIA1* locus may actually lead to a lower probability of target gene modification in the *NIA1*-edited chlorate resistant cells, so may not be desirable. In our view, at least as important as gRNA design is the need for the target gene locus to be accessible, for example by being transcriptionally active.

The use of ssODNs for SCREAM enables gene knockout, point mutation, the insertion of short protein sequences such as tags (Ferenczi et al. 2017) and should enable recombinase site insertion for subsequent recombinase-mediated gene knock-in (Li et al. 2020). Meanwhile, the demonstration by Kim et al. (2020) that up to 6 kb dsDNA incorporation could be directed using Cas9-mediated ds breaks (albeit with antibiotic selection to identify positive clones) suggests that precise knock-in of longer dsDNA via SCREAM should also be possible. We are currently investigating the efficiency of this process.

We expect the highly conserved nitrate reductase gene to be a suitable SCREAM marker for most microalgal species and we anticipate that the SCREAM approach will suffice for most routine CRISPR experiments to knock out or modify one or more endogenous genes, and to precisely insert transgenes at defined locations in the genome. All *NIA1^+^ Chlamydomonas* strains should be amenable, and some *NIA1^−^* strains (e.g. the *NIA1*-305 G-to-A point mutation; (Plecenikova et al. 2013)) might be readily repaired. Where *NIA1* repair is impossible, other dual-selectable markers genes such as *APRT* can be employed. While simplest to employ in haploid organisms, it can also be used in diploids (e.g. thale cress *Arabidopsis thaliana*) especially where selective mutation or artificial generation of haploids (Shen et al. 2023) can reduce the endogenous marker to a single copy.

## Materials and methods

### Cell culture

*Chlamydomonas reinhardtii* strains CC-400 and CC-1883 were obtained from the Chlamydomonas Resource Center (St Paul, MN, USA) and maintained on 1% (w/v) agar plates prepared with Tris-Acetate-Phosphate (TAP) medium (Gorman and Levine 1965). Liquid cultures were cultured in Erlenmeyer flasks (∼120 rpm shaking) under white fluorescent light (50 to 100 *µ*moles photons m^−2^ s^−1^). Prior to electroporation, cells were grown for several days in Nitrate Medium, comprising TAP lacking ammonium chloride, supplemented with 5 mm potassium nitrate to induce the *NIA1* locus. When required, 2 mm urea was provided to act as an alternative source of nitrogen for *NIA1^−^* cells.

### CRISPR guide RNAs

Guide RNAs (gRNAs) were designed using the Cas-Designer module of CRISPR RGEN Tools (Center for Genome Engineering, Institute for Basic Science, South Korea (http://www.rgenome.net/cas-designer/)). The parameters set in Cas-Designer for gRNA design were: up to 1,000 nucleotides of a region of a target gene, SpCas9 from *Streptococcus pyogenes* (PAM and RGEN type) and with *C. reinhardtii v5.0* as the target genome. The gRNA was selected after consideration of the target position, out-of-frame score, and the number of mismatches. Cas-OFFinder (http://www.rgenome.net/cas-offinder/) was used to check the proposed gRNA for possible off-site targeting. In addition, design recommendations by Wang et al. (2018) were taken into consideration. A list of gRNAs used in this work is provided in Table 1. Guide RNAs, tracrRNA and spCas9 enzyme were purchased from Integrated DNA Technologies as Alt-R CRISPR-Cas9 crRNAs. Strain names and phenotypes are given in lowercase italics (e.g. *stm6*, *arg7, cw15*). Gene nomenclature follows the Phytozyme gene names, with commonly used former names provided in brackets upon first use e.g. *NIA1 (Nit1)*.

### Single-stranded DNA oligonucleotides

The design of single-stranded DNA oligonucleotides (ssODNs) for homologous template directed repair was based on the relevant gene at the site of the anticipated DNA double-strand break created by *Streptococcus pyogenes* Cas9. All ssODNs for homology-directed repair were designed to include a ∼45 bp homology arm on either side of the ds break site as recommended by Wang et al. (2018) and Ferenczi et al. (2021). The ssODNs were unmodified DNA (*NIA1*) or had the end two base pairs modified with phosphorothiolate links for additional stability (*APRT*) and were purchased either from Integrated DNA Technologies or Sigma Aldrich (now Merck) and were rehydrated in nuclease free water to 100 *μ*M and stored at −20 °C. Sequences for the ssODNs employed here are given in Table 3.

### Cas9 RNP in vivo mix preparation

For each guide RNA, an RNP reaction was prepared, and in the case of experiments with mixed sets of gRNAs these were combined after RNP preparation at the time of electroporation. To prepare the reaction, tracrRNA and gRNA (Integrated DNA Technologies; IDT) were first mixed in IDT duplex buffer (30 mm HEPES, pH 7.5; 100 mm potassium acetate) and heated at 95 °C for five minutes. The solution was allowed to cool to room temperature to generate the RNA duplex. IDT duplex buffer and then Cas9 nuclease were added into the gRNA solution and incubated at room temperature for 15 min to allow RNP complex formation and then stored on ice. The final reaction contained, for each RNP used, 0.42 *μ*M Cas9, 0.8 *μ*M gRNA, 0.8 *μ*M tracrRNA, and (if used) 20 *μ*M ssODN. Where multiple gRNAs for a target gene were used, the total gRNA concentration was 0.8 *μ*M. Cas9-gRNA RNPs for each gene were made separately and combined just before the electroporation stage.

### Electroporation

Electroporation was conducted using a Bio-Rad Gene Pulser XCell equipped with Capacitance Extender (CE) and Pulse Controller (PC) modules and employed 2 mm or 1 mm gap cuvettes.

Reaction mixes comprised 50 × 10^6^ cells in 200 *µ*L Nitrate Medium with 40 mm sucrose, plus 50 *µ*L RNP mix (or IDT Nuclease-free Duplex Buffer). Initial electroporation employed 2 mm electrode gap cuvettes (Bio-Rad) at 450 V, 25 *µ*F, infinite resistance, exponential decay mode. The final scaled down protocol employed 5 × 10^6^ cells in 20 *µ*L plus 5 *µ*L RNP mix, electroporated with 1 mm gap electrodes at 200 V, 25 *μ*F, infinite resistance. After electroporation, cells were gently transferred to 10 mL Nitrate Medium for 48 h in low light (15 to 25 *µ*moles photons m^2^ s^−1^) to allow gene editing to occur, with 2 mm urea for forward (chlorate selection) experiments. For “nonelectroporated controls” the cells were transferred to an electroporation cuvette, then out to recovery medium without a pulse being applied.

### Clone selection

After recovery, cells were pelleted at 600 × *g*, and resuspended in a defined volume of Nitrate Medium. Dilutions were made into sterile Nitrate Medium so that ∼140 *µ*L was spread per plate. Selection plates (0.6% w/v agar) were prepared one day prior to plating with a Nitrate Medium + 2 mm urea base medium. Positive selection was accomplished with the inclusion of a selection agent, either 7.5 or 10 mm sodium or potassium chlorate (Sigma Aldrich), or 8 *µ*g mL^−1^ 2-fluoroadenine (FluoroChem). Negative (auxotrophic) selection was accomplished on plates containing 5 mm potassium nitrate but without urea. Colonies were picked with a sterile toothpick or pipette tip and placed in a suitable medium in 96-well plates for expansion.

### Image processing and colony scoring

Images were collected with an Olympus C5060WZ camera. The 9 cm plate image was separated into RGB channels using Adobe Photoshop (Adobe Systems Inc.) and a standard RGB setting was applied to enhance the contrast of the green colonies against the background (Red +185; Green −200, Blue +165) and images saved as monochrome images. For colony scoring, plate images were imported into ImageJ. The central region of the plate was isolated, and the outside (noncolony) part discarded. The contrast was set for maximum contrast between colonies and background and manually checked. A binary watershed filter was used to separate double colonies, and colonies larger than 16 pixels were quantified using the Analyse Particles function.

### Genomic DNA extraction

To yield high-quality double-stranded DNA, 5 mL of *C. reinhardtii* stationary phase-liquid culture was pelleted by centrifugation at 3000 × *g* for three minutes and resuspended in 500 *µ*L DNA extraction buffer, pH 9.5 (0.1 m Tris–HCl, 1 m KCl, and 0.01 m Na_2_EDTA). The cell suspension was frozen and thawed five times, incubated at 75 °C for 10 min, and cooled to room temperature. RNase A was added at a 10 *µ*g mL^−1^ final concentration and the sample incubated at 37 °C for one hour. DNA was then prepared by sequential extraction with 300 *µ*L phenol:chloroform:isoamyl alcohol (PCI; Sigma Aldrich) then chloroform:isoamyl alcohol (CI; Sigma Aldrich), precipitated with 70% (v/v) ethanol and resuspended in 100 *µ*L of 10 mm Tris, 1 mm EDTA (TE) buffer pH 8.0. The genomic DNA concentration was measured using a Nanodrop 2000c (Thermo Fisher Scientific, USA) checked by agarose gel electrophoresis and stored at −20 °C.

### Chelex-based genomic DNA extraction for PCR in 96-well plates

For single-stranded DNA suitable for rapid PCR analysis, the method of Cao et al. (2009) using Chelex resin was employed, including modifications described by Nouemssi et al. (2020) and Singh et al. (2018).

### Agarose gel electrophoresis

DNA was analysed using 1% (w/v) agarose gel electrophoresis at 75 to 100 V in TAE buffer (40 mm Tris; 20 mm acetic acid; 1 mm EDTA) with 1× SyberSafe dye (Thermo Fisher Scientific, USA) included. GeneRuler DNA 100 bp Ladder Mix (Thermo Fisher Scientific, USA) 3 *μ*L was typically employed as the marker, and DNA stained with SybrSafe dye (Thermo Fisher Scientific, USA) was visualized under UV light in a ChemiDoc XR system (Bio-Rad, USA).

### PCR amplification

Genomic DNA extracted from CC-1883 (*cw15*, *nit+*, *mt-*) was used as a DNA template for PCR using primers with annealing temperatures listed in Table 2. The PCR product was examined using agarose gel electrophoresis or used as the template for Cas9 in vitro digestion, mutation genotyping by restriction enzyme analysis, or for Sanger sequencing. Initial *NIA1* primers (Table 2; “A”) were used for Supplementary Fig. S1. All other experiments employed *NIA1* “B” primers which performed more efficiently.

### DNA sequencing

For Sanger sequencing, PCR products (3 *µ*L) were incubated with their respective primers (10 *µ*M) and sent to the Australian Genome Research Facility (AGRF, The University of Queensland, Brisbane, Australia). The resulting DNA sequence was analysed by pairwise and multiple sequence alignment using MUSCLE in SnapGene software (Dotmatics Limited) or by manual adjustment. Amino acid sequences were inferred and uploaded to Pfam (https://pfam.xfam.org/) for domain analysis. Amino acid sequences of mutated sequences were pairwise-aligned using BLASTP to identify altered amino acid residues.

### Cas9 RNP in vitro digestion

PCR amplicons from the *NIA1* gene of the first set of candidate knockout clones were evaluated for an intact Cas9 site by using Cas9-mediated digestion (Supplementary Fig. S1C). The in vitro digestion was prepared for two control reactions, a wild-type PCR amplicon combined with Cas9 only control (without gRNA), and gRNA only control (without Cas9). Amplicons from *NIA1* candidate knockout clones were then assessed using the RNP complex (1:2 ratio between Cas9 and gRNA). The ingredients for in vitro digestion were adapted from Ferenczi et al. (2017) and contained a preassembled RNP consisting of 600 nm Cas9 (IDT) and 1.2 *µ*M gRNA (IDT), 1.2 *µ*M tracrRNA (IDT), 1X NEB CutSmart Buffer (New England Biosciences), and 20 *µ*L of PCR product in a final volume of 25 *µ*L. The PCR product and NEB CutSmart Buffer were added and IDT Nuclease-free Duplex Buffer used to adjust the final volume to 25 *µ*L. All reactions were incubated at 37 °C for 45 min, followed by heat inactivation at 65 °C for 15 min. The fragments of the cleaved amplicons were separated using 1% (w/v) agarose gel electrophoresis and visualized using a Bio-Rad ChemDoc XR imager and Image Lab 4.1 software.

### Restriction enzyme screening of putative mutants

PCR amplicons were tested for the presence of specific restriction sites using restriction enzyme digestion. Restriction enzymes were purchased from New England Biolabs or Thermo Fisher and used according to the manufacturer's instructions. Digests were visualized by agarose gel electrophoresis.

### Accession numbers

The nucleotide sequences of target genes were obtained from the Phytozome plant genome database using the *C. reinhardtii* genome version 4 (*NIA1*), version 5.6 for *LHCBM1 (Cre01.g066917), LHCBM2 (Cre12.g548400)*, *LHCBM3 (Cre04.g232104), LHCA2 (Cre12.g508750), LHCA9 (Cre07.g344950)* and *MOC1 (Cre12.g542500; earlier name for STM6),* and the *C. reinhardtii* CC-4532 v6.1 genome for the *STM6 (Cre12.g542500_4532)*, and *APRT (Cre12.g548400_4532)* genes (https://phytozome.jgi.doe.gov/pz/portal.html).

## Supplementary Material

kiae427_Supplementary_Data

## Data Availability

The data underlying this article will be shared on reasonable request to the corresponding author.

## Supplementary data

The following materials are available in the online version of this article.

**Supplementary Appendix S1.** *NIA1* deletion using CRISPR/Cas9.

**Supplementary Appendix S2.** Chlorate selection.

**Supplementary Appendix S3.** Strategies to improve SCREAM efficiency.

**Supplementary Appendix S4.** Potential for off-target mutation in SCREAM-derived clones.

**Supplementary Figure S1.** CRISPR/Cas9 knockout of *NIA1* yields numerous chlorate-resistant mutants.

**Supplementary Figure S2.** Voltage titration with plasmid DNA.

**Supplementary Figure S3.** Voltage optimization of RNP electroporation.

**Supplementary Figure S4.** Test for chlorate resistance in colonies isolated from 2-fluoroadenine selection plates.

**Supplementary Figure S5.** Transformation with *APRT*-directed RNPs with or without *NIA1* induction.

**Supplementary Figure S6.** Sequence data for *APRT* PCR amplicons.

**Supplementary Figure S7.** Sequence data for *NIA1* PCR amplicons.

## Dive Curated Terms

The following phenotypic, genotypic, and functional terms are of significance to the work described in this paper:

nitrate CHEBI: CHEBI:17632

NIA1 Gramene: CHLRE_09g410950v5
NIA1 Araport: CHLRE_09g410950v5

APRT Gramene: CHLRE_17g704850v5
APRT Araport: CHLRE_17g704850v5

LHCBM2 Gramene: CHLRE_12g548400v5
LHCBM2 Araport: CHLRE_12g548400v5

MOC1 Gramene: CHLRE_12g542500v5
MOC1 Araport: CHLRE_12g542500v5

LHCBM1 Gramene: CHLRE_01g066917v5
LHCBM1 Araport: CHLRE_01g066917v5

LHCBM3 Gramene: CHLRE_04g232104v5
LHCBM3 Araport: CHLRE_04g232104v5

urea CHEBI: CHEBI:16199

chlorate CHEBI: CHEBI:49709

sodium chlorate CHEBI: CHEBI:65242

Nitrate reductase Gramene: AT1G77760
Nitrate reductase Araport: AT1G77760
