# Supplementary material for: A cyclical marker system enables indefinite series of oligonucleotide-directed gene editing in Chlamydomonas reinhardtii
Source: Plant Physiol. 2024 Aug 23;196(4):2330–45. doi: 10.1093/plphys/kiae427 (PMC11637769; doi:10.1093/plphys/kiae427)
Supplement: kiae427_Supplementary_Data [file kiae427_supplementary_data.zip › Supplementary Appendices S1-S4.pdf]

Plant Physiology

Supporting Information for

Strategy for unlimited cycles of scarless oligonucleotide directed gene editing in *Chlamydomonas reinhardtii*.

Ian L. Ross, Hong Phuong Le, Sabar Budiman, Dake Xiong, Fritz Hemker, Elizabeth A. Millen, Melanie Oey, and Ben Hankamer

Email: i.ross@imb.uq.edu.au, b.hankamer@imb.uq.edu.au

This PDF file includes:

Appendix S1 to S4 and supporting references

Supporting Information Text

Appendix S1. *NIA1* deletion using CRISPR/Cas9

Prior to using ssODN-directed mutation, we first confirmed that our chosen gRNAs (Table 1) successfully directed *NIA1* gene editing (Fig. S1) without the use of ssODNs. For this we employed the CC-1883 strain (CC-1883) which is derived from the basic 21 gr wild type *NIA1*$^{+}$ *Nit2*$^{+}$ *Chlamydomonas reinhardtii* Sager strain (now designated CC-1690) which can grow on nitrate.

After electroporation, candidate CRISPR-edited *NIA1*$^{-}$ mutants were identified by plating on NF-TAP plates supplemented with 2 mM urea and 10 mM potassium chlorate ($KClO_3$). Ammonium (including amino acid-derived ammonium obtained via extracellular deamination (Munoz-Blanco et al., 1990)) is a preferred nitrogen source for *Chlamydomonas*, but the presence of ammonium in the medium not only suppresses the nitrate import and assimilation system (including *NIA1*) but rescues *NIA1* mutants (Herrera et al., 1972; Hipkin et al., 1980). However *Chlamydomonas* can also utilise other organic nitrogen sources including urea (Munoz-Blanco et al., 1990), urate (Pineda and Cardenas, 1985) and purines (Pineda and Cardenas, 1996) which can be used to supply nitrogen to *NIA1* mutants without repressing the *NIA1* locus.

After seven days on selective plates, 871 colonies resulted from Cas9 RNP-transfected CC-1883, while 240 spontaneous mutant colonies arose from CC-1883 cells that were transfected without Cas9 RNP, for a ratio of 3.6:1 in favour of candidate CRISPR/Cas9 mutants. Representative plates are shown in Fig. S1A.

To eliminate spontaneous chlorate-resistant mutants among candidates from the RNP plates, colonies were randomly picked from the RNP^{+} plate and inoculated on both TAP plates and nitrate (NF-TAP+5 mM KNO_3) plates as described earlier, to observe their ability to utilise nitrate. Most picked colonies grew poorly on nitrate in comparison to the wild type control, but many showed partial growth which may have been due to the presence of organic nitrogen, for example from dead cells. Only colonies demonstrating a clear unambiguous failure to grow on nitrate were selected for further analysis. Forty-eight colonies were found to definitively lack the ability to grow on nitrate (Fig. S1B), thus these mutants were subjected to *NIA1* genotyping.

Genomic DNA of suspected *NIA1*$^{-}$ mutants was extracted and used for PCR (Table 2; *NIA1* B primers) to amplify the gRNA targeted region from *NIA1*. To test for indels at the gRNA site, the *NIA1* PCR product was treated with Cas9 in vitro digestion. Wildtype amplicons were observed to be cleaved at the gRNA site generating two amplicon bands around 500bp and 200bp. In contrast, mutated *NIA1* amplicons were expected to be uncut (~704 bp). Twenty-three candidate clones that failed to cut with Cas9 digestion were then sequenced using the PCR primers as sequencing primers.

Sanger sequencing successfully identified all 23 candidate mutants as presumptive Cas9-induced mutants (Fig. S1d). This is a minimum, as amplicons that were successfully digested with Cas9 could still have contained mutations that did not prevent gRNA binding to the template. The number of altered nucleotides ranged from 1 to 28 nucleotides for deletion and 1 to 59 bases for insertion. Five mutants, 45, 58, 66, 69 and 79, had indels that restored the reading frame of the *NIA1* gene. In the case of mutant 79, only two amino acids were deleted. Despite this, they all grew poorly on nitrate as a sole nitrogen source suggesting that even small changes in the moco binding site (exon 2) were disruptive to *NIA1* function. Of interest for later experiments, out of the 23 sequenced mutants, only one lost the *Pfl*MI restriction site.

Appendix S2. Chlorate selection

1. Spontaneous chlorate resistant mutants: Chlorate is imported via the nitrate import system and can be converted by NR to the toxic metabolite chlorite (ClO_2^-) which was originally thought to be the main route of chlorate toxicity in *Chlamydomonas* (Prieto and Fernandez, 1993). Mutations in a number of genes can lead to chlorate resistance, including mutations in nitrate (Quesada and Fernandez, 1994) and molybdate transporters (Tejada-Jimenez et al., 2007), moco synthesis (*Nit3*-*Nit7*; (Aguilar et al., 1992; Galvan et al., 1992)) and regulators of *NIA1* expression such as *Nit2* (Fernandez and Matagne, 1986; Galvan et al., 1992). Spontaneous chlorate resistant mutants are always present at a low level in any *Chlamydomonas* population. In our experience these are usually present at a much lower level than the RNP-mediated mutants in *NIA1*, but their presence must always be assumed. The level of these spontaneous mutants can be assessed by comparison of RNP transfections to control plates on which a similar number of cells have been spread, or, in the case of a CRISPR experiment, cells which have been electroporated without RNPs. Efficient CRISPR experiments greatly increase the ratio of CRISPR-generated *NIA1*⁻ clones to spontaneous mutants and in practice we have not found this to be a significant obstacle.

2. Potential mutagenicity from chlorate: Prieto and Fernandez (Prieto and Fernandez, 1993) treated cells in solution with chlorate for 20-24h and plated the survivors on minimal media, demonstrating that chlorate at high concentrations (15-100 mM) can be mutagenic, however they also showed that competence for nitrate uptake is absolutely required for mutagenicity. This suggests that knockout of *NIA1* would not necessarily prevent chlorate uptake, leaving a risk of chlorate mutagenicity during selection. However, Navarro et al. (Navarro et al., 1996) subsequently identified a key role for NR enzyme activity in nitrate (and thus chlorate) uptake and showed that the loss of NR activity downregulates chlorate import, suggesting that failure to import chlorate is actually the primary basis of chlorate resistance in *NIA1*⁻ cells.

We considered the possibility that chlorate toxicity can also lead to mutagenicity, which would be undesirable during CRISPR gene editing. While colonies showing *spontaneous* chlorate resistance rarely contain *NIA1* inactivation (Prieto and Fernandez, 1993), targeted *NIA1* inactivation via CRISPR definitely produces chlorate resistance. In the SCREAM protocol, *NIA1* mutants are generated during the CRISPR editing phase (overnight in the dark, following electroporation), and

do not experience chlorate selection until after plating out at 16-48h. At this stage, *NIA1* editing means that successfully CRISPR-edited cells will lack or have low levels of nitrate reductase, resulting in down-regulated chlorate uptake transport (thereby limiting uptake of chlorate) and will also be unable to convert chlorate to chlorite. We therefore conclude that *NIA1* mutants, by the time of plating on selective media, will likely not be susceptible to chlorate mutagenesis. Until this question is definitively settled, we used the lowest feasible concentration of chlorate that enables effective and reliable selection (10 mM) and do not routinely maintain mutants on chlorate containing media.

Appendix S3. Strategies to improve SCREAM efficiency

1. Time required for CRISPR editing.

Because CRISPR editing takes many hours, there is a tradeoff involved in the length of time that mutants are left prior to plating on selective media with more mutants expected if cells are left for longer before plating on selective media. Furthermore, on chlorate media, time is required for the intrinsic levels of NR protein to decay before selection is possible. On the other hand, long periods prior to recovery allow cell replication, resulting in multiple colonies from single edited cells. To accurately gauge clone numbers, we initially kept the time between electroporation and plating relatively short (16-24h) and did not identify any clones with identical indels among those we sequenced. However, for users simply interested in obtaining edited genes, the risk of picking duplicate clones is small because of the vast number of colonies typically generated, we recommend leaving reactions for at least 48h to maximise the chances of gene editing prior to plating, while also minimizing any risk of NR-dependent chlorate mutagenesis.

2. Use of multiplex gRNAs during a single SCREAM stage: We have successfully used multiple gRNAs and ssODNs to enable multiple target gene edits per cycle. Usually however, the actual electroporation and plating for a given SCREAM stage is much less work than screening for complex target gene mixtures. Consequently, it is usually faster to conduct the next sequential round of SCREAM than to identify more complex patterns of gene editing in a single round. As standard practice, therefore, we aim at a single target gene per stage. For illustration, if true co-targeting efficiency is 20%, then the number of clones that need to be examined for a 50% chance of obtaining a fully edited clone is 5 clones for a single target gene, 25 for two genes, and 15,625 for five genes. As the co-targeting efficiency rises, the feasibility of multiplexing increases, but consistently high efficiencies are required for all genes for this strategy to work. In comparison, with successive rather than multiplexed SCREAM, there is a high probability of obtaining a successfully edited clone with a fixed, relatively small amount of work, and these clones can then be used in parallel to create intermediate combinations of all possible edits with a high degree of certainty.

The exception is where a more effective screening strategy than PCR is available for the edited target genes, such as a fluorescent signal selectable by flow cytometry, or where cells with a

specific phenotype are sought, which can be picked using independent criteria (e.g. pale green colonies) and interrogated for their genotype. Multiple edits per SCREAM stage may also be useful where a combinatorial library of edited cells is required and high target gene conversion is known to occur (e.g. highly transcriptionally active genes). Multiplexing approaches that require *in situ* generation of gRNAs via transient transfection do not seem to be a promising approach in *Chlamydomonas* due to the low efficiency of both transient transfection and CRISPR conversion in a given cell population.

3. *NIA1*:Target gRNA ratio: Another strategy is to bias the amounts of gRNAs for the *NIA1* compared to the target gene, for example by 1:10. Although total chlorate resistant colonies may drop, the vast excess of chlorate-resistant colonies even with 5 million cells, means that many more clones would be isolated than are required, further reducing screening requirements. Offsetting this, longer incubation prior to selection should increase total CRISPR conversion rates (see B above). The use of "suboptimal" *NIA1* gRNAs may also improve the *NIA1*:target ratio.

4. Multiplex PCR screening and clone pooling: PCR analysis readily identifies large deletions in the PCR amplicon, but subtle mutations are more time consuming to identify. The use of fluorescent labelled primers, and primers designed specifically against the mutant site (if possible) can simplify the PCR screening of colonies both for the *NIA1* gene (e.g. correct incorporation of the ssODN in the chlorate selection stage) and for the target gene (again, if a specific ssODN sequence is being inserted). Clones that appear to yield correct amplicons by mutation-specific PCR can then be checked by restriction digestion so that only clones with a high likelihood of being correct need be sequenced. If a mutation appears to be present at an unusually low frequency, pooling clone DNA can be used to reduce the number of PCR reactions required.

Appendix S4. Potential for off-target mutation in SCREAM-derived clones

In *Chlamydomonas*, dsDNA breaks are mainly repaired using Ku70/Ku80 mediated non-homologous end-joining (NHEJ) which typically creates indels or polymerase theta-mediated end joining (TMEJ; also known as microhomology mediated end joining or MMEJ) which is the main route for ssDNA incorporation, though both processes may act at a given site (Ferenczi et al., 2021; Sizova et al., 2021). There are several ways that unintended mutations could be created during our experimental process, including (a) Cas9 RNP-directed double strand breaks at sites other than the target region (off-target cleavage), (b) NHEJ/TMEJ-mediated, inappropriate repair or insertion of ssODNs at the intended co-targeted sites created by Cas9, (c) homology-directed insertion of a ssODN by non-Cas9-mediated HDR (e.g. homologous recombination), and (d) insertion of indels or ssDNA at random double strand breaks by NHEJ/TMEJ. All have the potential to create mutants with unintended mutations in addition to those that have been verified.

(a) Cas9 RNP-directed double strand breaks at off-target sites.

Baek et al. (2018) identified 11 sites in the *Chlamydomonas* genome that differed by 4 nucleotides from their gRNA, using deep sequencing of those loci to identify off-target mutations. None were found, suggesting that the use of electroporated RNPs, present only transiently in the cell, is unlikely to generate off-target mutations in cases where the gRNA differs significantly from the genome sequence. In our case, we used Cas-OFFinder (see doi.org/10.1093/bioinformatics/btu048) to design the gRNA and avoid off-target sites by ensuring no similar sites with less than 4 nt differences from the chosen gRNA (Bae et al., 2014). Importantly, Cas-9 directed mutations are unlikely due to the *NIA1* ssODN because (from BLAST searching) there is no genomic region with sufficient homology to the relevant *NIA1* gRNA sequence to create breaks at other loci. Of course, each new target gene gRNA is unique and will need to be examined for this possibility.

(b) NHEJ/TMEJ-mediated insertion of ssODNs at the co-targeted sites created by Cas9.

Since any gRNA-directed, Cas9-mediated ds break is a target for NHEJ repair potentially with subsequent indel formation, insertion of a provided ssODN intended for one site (e.g. *NIA1*) might occur at a different (target) site, for example via microhomology. In our experiments, the most likely sites of unintended DNA insertion are at the double strand breaks that are being created deliberately and frequently by using gRNA-directed Cas9 (i.e. insertion of the ssODN for *NIA1* at the co-targeted *APRT* locus). Since both loci are checked before a cell line is used for experiments, this would be readily detected. So far we have only twice seen insertion of ssODN sequence at non-homologous break sites (and these may in fact have arisen from genomic DNA released from the ds break site during repair rather than the ssODN) which leads us to believe that such insertion is uncommon, though certainly possible (a similar issue arises when considering the role of spontaneous point mutations in clones isolated from single colonies).

(c) insertion of a ssODN by non-Cas9-mediated HDR

The use of ssODN to effect mutations without CRISPR has been demonstrated in *Chlamydomonas* by using large amounts of ssODNs (40 μg per electroporation) and targeting the selectable acetolactate synthase gene (Jiang et al., 2017). We do not, at present, have sufficient data to understand the mechanism of this mutation. However, the frequency of successful mutation was only around 1 in 10^7 suggesting that this is an inefficient process. Therefore we expect ssODN-directed mutation of either the homologous target site or other partly homologous sites to occur rarely if at all.

(d) insertion of ssDNA at random double strand breaks via NHEJ/TMEJ.

Chlamydomonas is known to randomly insert dsDNA at double strand breaks in the genome, which is the basis of transfection experiments with dsDNA plasmid vectors, and which occurs during deliberate transfection experiments at a frequency of up to 1 in 10^5. The insertion of non-homologous ssDNA at random genomic double strand breaks is not as well described, but it is

possible. This possibility exists whenever DNA of any kind is transfected into a cell which simultaneously contains a random double strand break and which is undergoing NHEJ/TMEJ repair (in which case a mutation at the site is likely in any case).

Precautions against unintended mutations

In phenotyping experiments with the mutants created using SCREAM, we use a set of independent mutant clones (typically 3-6) to check that a uniform phenotype is observed, since it is unlikely that off-target effects would be identical from clone to clone. It should be noted that since most *Chlamydomonas* CRISPR mutations are used for phenotypic experimental analysis (rather than for, say, therapeutic utility in humans) the presence of off-target mutations in some *Chlamydomonas* clones does not necessarily prevent mutant lines from being useful.

We considered using Southern blotting to try to identify whether the mutant ssODN is present at only one location in the genome (i.e. at the *NIA1* and/or target locus, for example when an *APRT* ssODN is used). However, potential fragmentation and rearrangement of the ssODN during insertion (typically observed during plasmid insertion into the *Chlamydomonas* genome) means that Southern blotting can detect, but cannot yield a confident exclusion of off-target mutations. Ultimately, whole genome sequencing is the only way to identify whether an isolated clone is identical to the parent starter cell line. This is not practical when hundreds of clones are being generated, but it would be desirable for clones that are to be used as reference points for a significant amount of ongoing work.

Appendix S1-S4 References

Aguilar MR, Prieto R, Cardenas J, Fernandez E (1992) Nit-7 - a new locus for molybdopterin cofactor biosynthesis in the green-alga *Chlamydomonas reinhardtii*. Plant Physiology **98:** 395-398

Bae S, Park J, Kim JS (2014) Cas-OFFinder: a fast and versatile algorithm that searches for potential off-target sites of Cas9 RNA-guided endonucleases. Bioinformatics **30:** 1473-1475

Baek K, Yu J, Jeong J, Sim SJ, Bae S, Jin E (2018) Photoautotrophic production of macular pigment in a strain generated by using DNA-free CRISPR-Cas9 RNP-mediated mutagenesis. Biotechnology and Bioengineering **115:** 719-728

CC-1883. *In* The Chlamydomonas Resource Center, St Paul MN, p www.chlamycollection.org

Ferenczi A, Chew YP, Kroll E, Von Koppenfels C, Hudson A, Molnar A (2021) Mechanistic and genetic basis of single-strand templated repair at Cas12a-induced DNA breaks in *Chlamydomonas reinhardtii*. Nature Communications **12:** 6751

Fernandez E, Matagne RF (1986) In vivo complementation analysis of nitrate reductase-deficient mutants in *Chlamydomonas reinhardtii*. Current Genetics **10:** 397-403

Galvan A, Cardenas J, Fernandez E (1992) Nitrate reductase regulates expression of nitrite uptake and nitrite reductase activities in *Chlamydomonas reinhardtii*. Plant Physiology **98:** 422-426

Herrera J, Barea JL, Paneque A, Losada M, Maldonado JM (1972) Regulation by ammonia of nitrate reductase synthesis and activity in *Chlamydomonas reinhardtii*. Biochemical and Biophysical Research Communications **48:** 996-+

Hipkin CR, Albassam BA, Syrett PJ (1980) The roles of nitrate and ammonium in the regulation of the development of nitrate reductase in *Chlamydomonas reinhardtii*. Planta **150:** 13-18

Jiang WZ, Dumm S, Knuth ME, Sanders SL, Weeks DP (2017) Precise oligonucleotide-directed mutagenesis of the *Chlamydomonas reinhardtii* genome. Plant Cell Reports **36:** 1001-1004

Munoz-Blanco J, Hidalgo-Martinez J, Cardenas J (1990) Extracellular deamination of l-amino-acids by *Chlamydomonas reinhardtii* cells. Planta **182:** 194-198

Navarro MT, Prieto R, Fernandez E, Galvan A (1996) Constitutive expression of nitrate reductase changes the regulation of nitrate and nitrite transporters in *Chlamydomonas reinhardtii*. Plant Journal **9:** 819-827

Pineda M, Cardenas J (1985) The urate uptake system in *Chlamydomonas reinhardtii*. Biochimica Et Biophysica Acta **820:** 95-99

Pineda M, Cardenas J (1996) Transport and assimilation of purines in *Chlamydomonas reinhardtii*. Scientia Marina **60:** 195-201

Prieto R, Fernandez E (1993) Toxicity of and mutagenesis by chlorate are independent of nitrate reductase-activity in *Chlamydomonas reinhardtii*. Molecular & General Genetics **237:** 429-438

Quesada A, Fernandez E (1994) Expression of nitrate assimilation related genes in *Chlamydomonas reinhardtii*. Plant Molecular Biology **24:** 185-194

Sizova I, Kelterborn S, Verbenko V, Kateriya S, Hegemann P (2021) Chlamydomonas POLQ is necessary for CRISPR/Cas9-mediated gene targeting. G3-Genes Genomes Genetics **11**

Tejada-Jimenez M, Llamas A, Sanz-Luque E, Galvan A, Fernandez E (2007) A high-affinity molybdate transporter in eukaryotes. Proceedings of the National Academy of Sciences of the United States of America **104:** 20126-20130
