# Supplementary material for: A cyclical marker system enables indefinite series of oligonucleotide-directed gene editing in Chlamydomonas reinhardtii
Source: Plant Physiol. 2024 Aug 23;196(4):2330–45. doi: 10.1093/plphys/kiae427 (PMC11637769; doi:10.1093/plphys/kiae427)
Supplement: kiae427_Supplementary_Data [file kiae427_supplementary_data.zip › Supplementary Figures S1-S7.pdf]

Plant Physiology

Supporting Figures for

A cyclical marker system enables indefinite series of oligonucleotide-directed gene editing in *Chlamydomonas reinhardtii*

Ian L. Ross, Hong Phuong Le, Sabar Budiman, Dake Xiong, Fritz Hemker, Elizabeth A. Millen, Melanie Oey, and Ben Hankamer

Email: i.ross@imb.uq.edu.au, b.hankamer@imb.uq.edu.au

This PDF file includes:

Figures S1 to S7

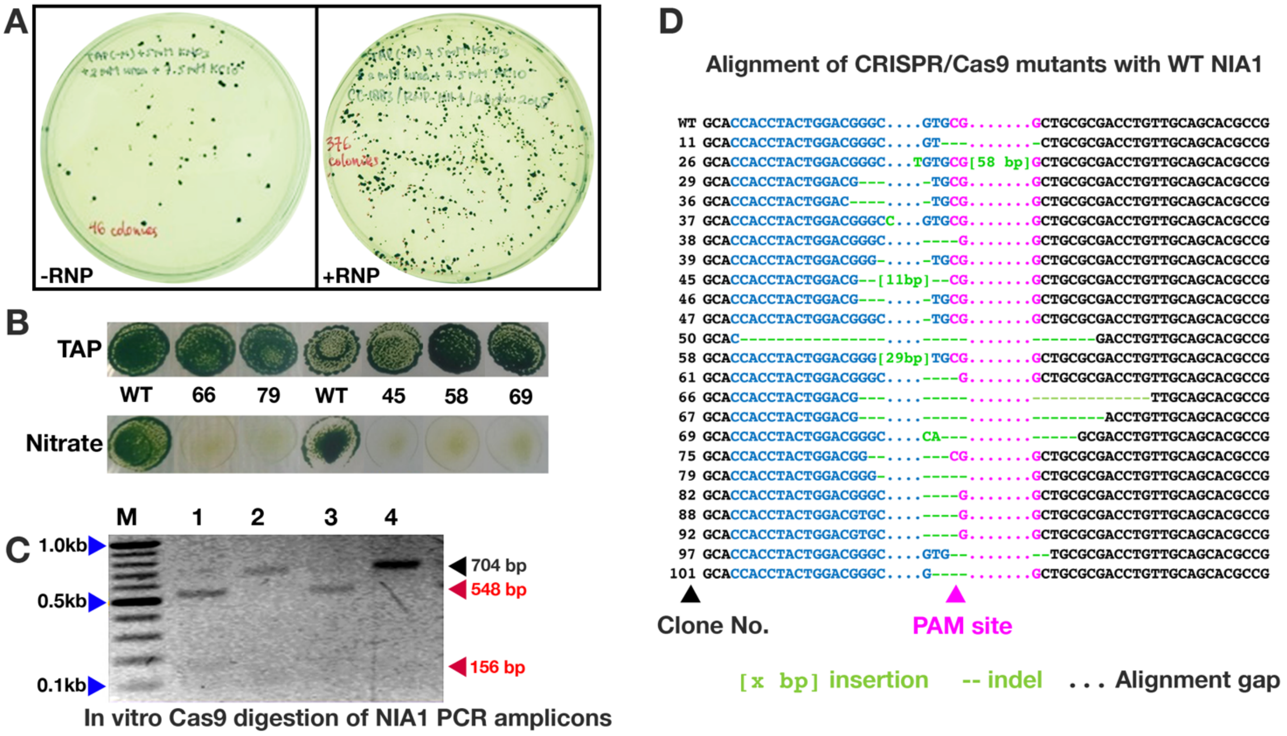

Supplementary Figure S1. CRISPR/Cas9 knockout of *NIA1* yields numerous chlorate-resistant mutants. **A:** Representative plates spread with electroporated cells without (left) and with (right) anti-*NIA1* RNPs demonstrates that RNPs lead to numerous colonies (871 total) compared to spontaneous chlorate resistant mutants (240 total). Images were digitally extracted for comparison and the contrast adjusted as a composite image. **B:** The use of nitrate vs TAP plates identifies colonies that fail to grow on nitrate as a sole nitrogen source. Two WT controls and 5 mutants are included for illustration. **C:** PCR amplification of the 704 bp *NIA1* target region (in the wt), followed by in vitro Cas9 digestion to 548bp and 156 bp fragments, directed by gRNA against the *NIA1* target sequence. Clones (red asterisks) in which indels have disrupted the target site are not cleaved, yielding a single ~700 bp band. **D:** Wildtype and mutant sequences (Clones 11-101) across the *NIA1* target region, aligned to show gRNA site (blue), PAM site (magenta text), deleted bp (pale green dashes) and position of 11bp, 29bp and 58bp inserts (pale green text) in clones 45, 58 and 26 respectively.

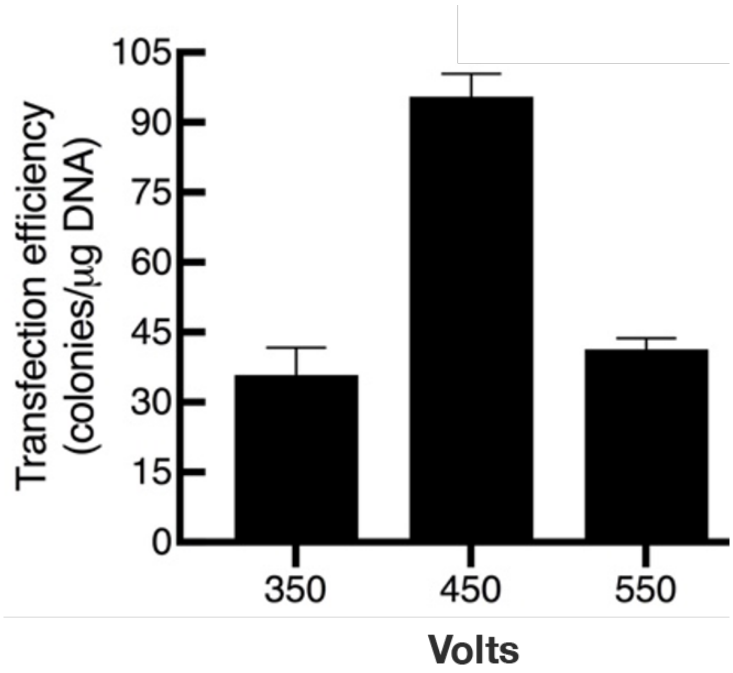

Supplementary Figure S2. Voltage titration with plasmid DNA. CC-1883 cells (50 million) were resuspended in a 2 mm gap cuvette in ToS (TAP medium with 40 mM sucrose) containing 2 μg of *Sac*I-linearised plasmid DNA (in-house Bluescript-based plasmid pMO48 which carries the *aph*VIII gene for paromomycin resistance). Electroporation was conducted using an exponential decay pulse with a range of voltage values (350 V, 450 V and 550 V), infinite resistance and 25 μF capacitance. The generated transformants were selected on 1.5% TAP agar plates supplemented with 15 μg mL^{-1} of paromomycin. Transformant colonies appeared after 5-7 days and were counted.

The transfection efficiency was defined as the number of colonies forming units per microgram plasmid DNA (CFU μg^{-1}). Data shows mean +/- 1 standard deviation from 3 biological replicates. A control electroporation without plasmid, and the non-electroporated control (plasmid included but no pulse) showed no colonies on the TAP agar plates in the presence of paromomycin (data not shown).

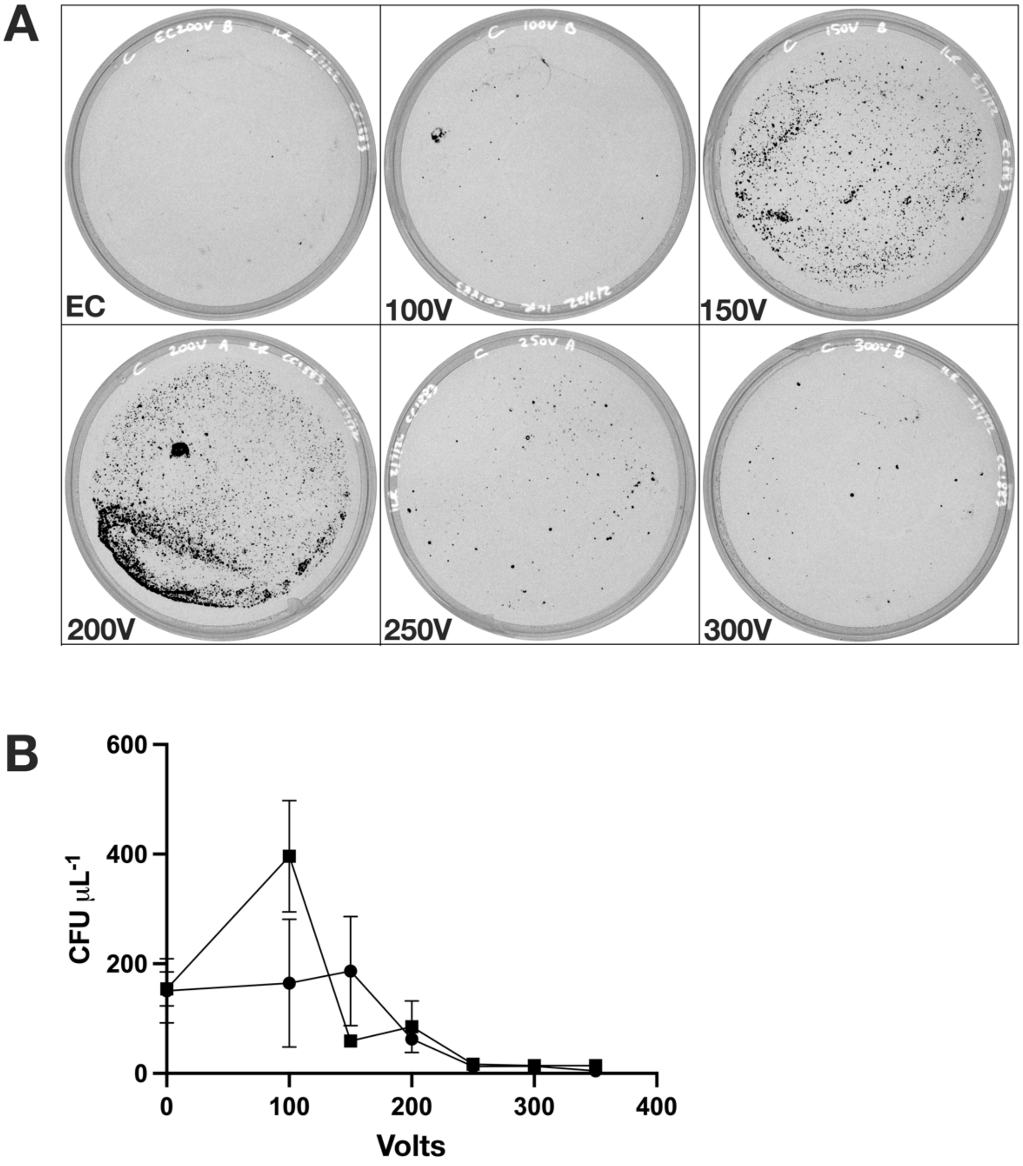

Supplementary Figure S3. Voltage optimization of RNP electroporation. **A:** CRISPR edited plates at different voltages; representative plates showing the yield of chlorate-resistant colonies vs electroporation voltage (V). The *NIA1* gRNA and *NIA1*-targeting ssODN was used with selection on chlorate media, to identify transformants that had successfully undergone disruption of the *NIA1* locus as indicated by chlorate resistance. Representative example plates are shown. Electroporation control (EC) was electroporated at 200V but without RNPs. **B:** Cell death induced by electroporation; estimation of colony forming units (CFU) per µL of electroporated cell suspension by plating on TAP agar. Five million cells, electroporated and resuspended in 10 mL of recovery medium were sampled on (●) Day 1 (12 hours after electroporation) and (■) Day 2 (just before plating on selective media). Data shows mean +/- 1 standard deviation. Plates were photographed at the same time and processed as described in the Methods and Materials section ("Image processing and colony scoring"). Images were digitally extracted for comparison.

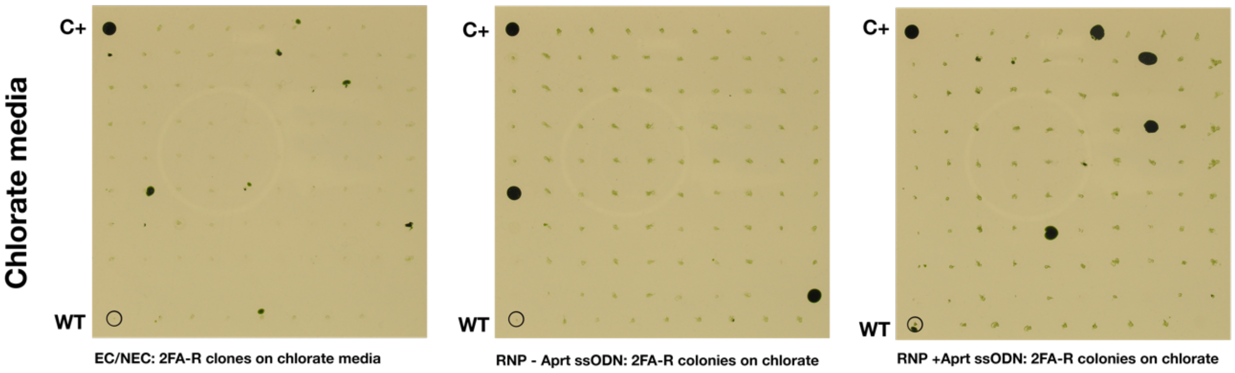

Supplementary Figure S4. Test for chlorate resistance in colonies isolated from 2-fluoroadenine selection plates. Resistant colonies from 2-fluoroadenine plates (i.e. presumed *APRT* disrupted) were re-gridded on chlorate selection to identify *APRT* candidate mutants that may also have *NIA1* mutations. Pinprick sized colonies on the control plate are likely due to spontaneous mutations in multiple genes of the nitrate assimilation pathways which confer partial chlorate resistance (as seen also in the "WT" control in panel 3). Control "C+" is a clone resistant to 2FA (top row) or chlorate (bottom row). Left panel (EC/NEC): colonies isolated from control plates lacking RNPs. Middle panel: colonies isolated from plates employing RNPs made from dual gRNAs, and no *APRT*-directed ssODN. Right panel: colonies isolated from plates employing RNPs made from a single gRNA and the corresponding *APRT*-directed ssODN.

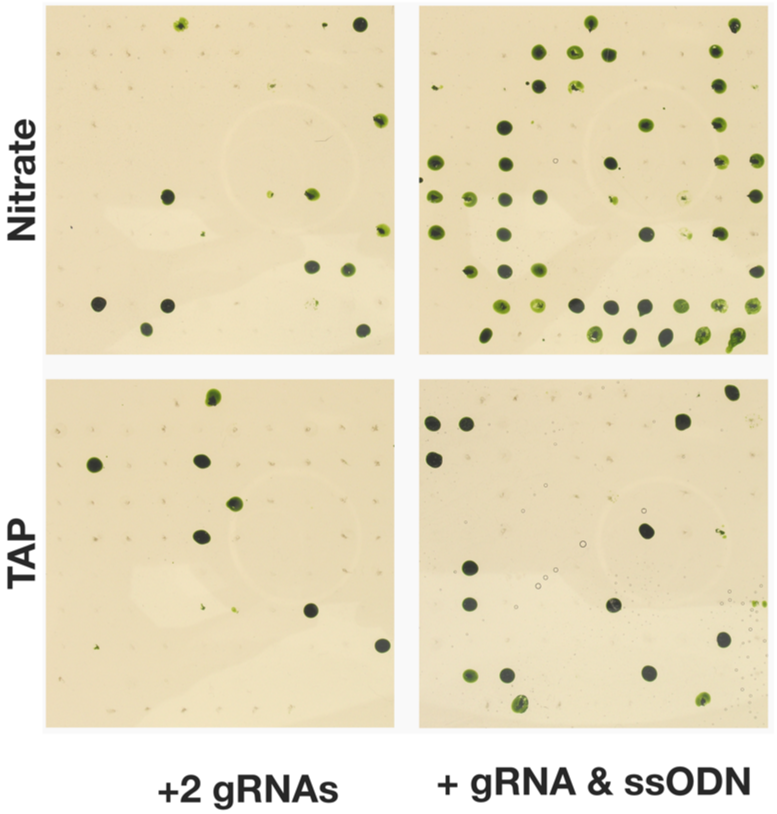

Supplementary Figure S5. Transformation with *APRT*-directed RNPs with or without *NIA1* induction. Co-targeting of the *APRT* gene from chlorate-resistant (presumptive *NIA1*-mutant) colonies was estimated in cells grown in Nitrate Medium (*NIA1* induced) or TAP (*NIA1* repressed) media prior to electroporation. Following electroporation of CC-1883 cells with *NIA1* RNP, anti-*NIA1* ssODN, and either 2 *APRT* RNPs or a single *APRT* gRNA plus an *APRT*-directed ssODN, 96 chlorate resistant colonies were picked from selection plates, grown in TAP media and spotted onto 2-fluoroadenine-containing square plates. The top row shows chlorate resistant colonies from cells that were electroporated after growth in nitrate medium, conditions which induce nitrate reductase. Co-targeting of *APRT* (including other colonies not shown here) was 11.7% with dual gRNAs, 41.6% with ssODN. The bottom row shows chlorate resistant colonies from cells that were electroporated after growth in TAP medium, in which the presence of ammonium is known to repress the *NIA1* gene. Co-targeting of *APRT* was 8.3% with dual gRNAs, 14.2% with ssODN. Although the repression of the *NIA1* gene was expected to reduce the chances of *NIA1* editing relative to *APRT*, the greatest level of *APRT* editing was seen under standard conditions (i.e. growth in Nitrate Medium). However, the level of *APRT* gene activation during growth in TAP is unknown. Further work is needed to identify the relationship between co-targeting and gene activation.

Condition	Phenotype	Colony	130bp core APRT sequence alignment	Insert/deletion sequence
WT genomic			TCGTGGCATTCCTGACTTTCCCCACAAGGGTATTCTGTTCTGGGATGTCACCACCATCATGCTGAACCACCAGGCGTTCCAGTACAGCATTGACCTGTTCGCTGAGCAGTACAAGGACAAGAAGATTGAC	
Nit(+)	2-FA resistant	N21	TCGTGGCATTCCTGACTTTCCCCACAAGGGTATTCTGTTCTGGGATGTCACCACCATCATGCTGAACCACC-----[del 452 bp]------------------------------------	452 nt deletion
Nit(+)	2-FA resistant	N31	TCGTGGCATTCCTGACTTTCCCCaCAAGGGTATTCTGTTCTGGGATGTCACCACCATCATGCTGACC-ACCAGGAT---CAGTACAGCATTGACCTGTTCGCTGAGCAGTACAAGGACAAGAA	1 nt and 3 nt deletions, ssODN site partially altered to match ssODN
Nit(+)	2-FA resistant	N37	Inserted sequence from *NIA1* exon 2: CGCGTGCGGCTGCGCGACCTGTTGCAGCACGCCGGCGTTCCAGTACAGCATTGACCTGTTCGCTGAGCAGTACAAGGACAAGAA	Insert from *NIA1 (ssODN or gDNA?)*
Nit(+)	2-FA resistant	N47	TCGTGGCATTCCTGACTTTCCCCACAAGGGTATTCTGTTCTGGGATGTCACCACCATCATGCTGAACCACCAG-TGAT-CAGTACAGCATTGACCTGTTCGCTGAGCAGTACAAGGACAAGAA	ssODN correctly inserted
Nit(+)	2-FA resistant	N51	TCGTGGCATTCCTGACTTTCCCCACAAGGGTATTCTGTTCTGGGATGTCACCACCATCATGCTGAACCACCAC-----[84 bp ins]------TGTTCGCTGAGCAGTACAAGGACAAGAA	22 nt deletion, 84 nt insertion (N51 insert, below)
Nit(+)	2-FA resistant	N67	TCGTGGCATTCCTGACTTTCCCCACAAGGGTATTCTGTTCTGGGATGTCACCACCATCATGCTGAACCACCAGG-[199 bp ins]-ATTGACCTGTTCGCTGAGCAGTACAAGGACAAGAA	14 nt deletion, 199 bp insertion
Nit(+)	2-FA resistant	N74	TCGTGGCATTCCTGACTTTCCCCACAAGGGTATTCTGTTCTGGGATGTCACCACCATCATGCTGAACCACCAG-TGAT-CAGTACAGCATTGACCTGTTCGCTGAGCAGTACAAGGACAAGAA	ssODN correctly inserted
Nit(+)	2-FA resistant	N82	TCGTGGCATTCCTGACTTTCCCCACAAGGGTATTCTGTTCTGGGATGTCACCACCATCATGCTGAACCACCAGG-59bpCAGTACAGCATTGACCTGTTCGCTGAGCAGTACAAGGACAAGAA	5 nt deletion, 59 bp insert (N82 insert sequence below)
TAP(+)	2-FA resistant	T2	[47 nt ins]----------------GGGTATTCTGTTCTGGGATGTCACCACCATCATGCTGAACCACCAG-TGAT-CAGTACAGCATTGACCTGTTCGCTGAGCAGTACAAGGACAAGAA	2 copies of ssODN inserted, one complete, one incomplete
TAP(+)	2-FA resistant	T8	TCGTGGCATTCCTGACTTTCCCCACAAGGGTATTCTGTTCTGGGATGTCACCACCATCATGCTGAACCACCAGGCGTTCCAGTACAGCATTGACCTGTTCGCTGAGCAGTACAAGGACAAGAA	G inserted at orange nt
TAP(+)	2-FA resistant	T13	TCGTGGCATTCCTGACTTTCCCCACAAGGGTATTCTGTTCTGGGATGTCACCACCATCATGCTGAACCACCAGGCGTTCCAGTACAGCATTGACCTGTTCGCTGAGCAGTACAAGGACAAGAA	G inserted at orange nt
TAP(+)	2-FA resistant	T24	TCGTGGCATTCCTGACTTTCCCCACAAGGGTATTCTGTTCTGGGATGTCACCACCATCATGCTGAACCACCAGC-GTATCAGTACAGCATTGACCTGTTCGCTGAGCAGTACAAGGACAAGAA	Incomplete insertion of ssODN
TAP(+)	2-FA resistant	T43	TCGTGGCATTCCTGACTTTCCCCACAA-[69 bp ins]-TGGGATGTCACCACCATCATGCTGAACCACCAG-TGAT-CAGTACAGCATTGACCTGTTCGCTGAGCAGTACAAGGACAAGAA	3 copies of ssODN inserted.
TAP(+)	2-FA resistant	T50	TCGTGGCATTCCTGACTTTccccaCAAGGGTAtTCtgTTCTGGGATGTCACCACCATCATGCTGAACCACCAGGCgTTccagtacAGCATTGACCTGTTCGCTGAGCAGTACAAGGACAAGAA	A inserted at 5' orange nt, GG inserted at 3' orange nt
TAP(+)	2-FA resistant	T81	TCGTGGCATTCCTGACTTTCCCCACAAGGGTATTCTGTTCTGGGATGTCACCACCATCATGCTGAACCACC-GGCGTTCCAGTACAGCATTGACCTGTTCGcTGAGCAGTACAAGGACAAGAA	A deleted

Samples found to possess wildtype sequence are not included, but are noted below

Key: **Dark green text** = point mutations; **Orange nt** = site of insertion; **Light blue text** = insert sequence; **Plum text = inversions**; dashes = deleted nt, with insert length if deleted nt were replaced by an insert; Ambiguous nt = N
Wt genomic sequence at top incudes PAM (magenta) gRNA (dark blue) and site of ssODN sequence (light green). Correct insertion of ssODN marked by blue shading

Sequence of inserts:

Clone	Insert sequence
**N51**	CTGAGCATTGACCTGTTCGCTGTACTGGCGTTCCAGTACAGCATTGACCTGTTCGCTGAGCAGTACAAGGACAAGAAGATTGAC - teal derived from genomic or ssODN, pink derived from ds DNA genomic
**N82**	GTATTCTGTTCTGGGATGTCACCACCATCATGCTGTACTGATCACTGGTGGTTCAGCGGCGTTC (59bp insert sequence)
**T2**	atcgtGGCATTCTTGATAATGGTGGTTATTCTGTTCTTGATAATGGT (47 nt insert in clone T2)
**T43**	gtATTCTGTTcTGGGATGTCACCACCATCATGcTGAACCACCAGTGATCAGTACAGCATTGaCcTGTTC = 69 bp net insert sequence

Notes concerning clones that were not sequenced:
2FA resistant clones are candidate APRT mutants.
• No clear amplicon band (i.e. loss of primer site) was observed by PCR for clones N9, N16 and N60
• Large amplicon insertions were evidenced by PCR for clones T1 and T66
A large deletion was evidenced by PCR for clone N21 (and confirmed by sequencing)
Wildtype sequence was obtained for 2FA-resistant clones N27, N90, T62, T84, T86, T87, T91, T95
(these clones may have mutations elsewhere in the APRT gene)

2FA sensitive clones were assumed to have wildtype APRT.
Wildtype sequence was obtained for 2FA-sensitive clones T6, T11, T16, T22, T28, T35, T42, T46, T49, T56
Wildtype amplicons were obtained (but not sequenced) from 2FA sensitive clones N11, N23, N32, N35, N36, N44, N59, N65, N66 and N79
(given their 2FA sensitive phenotype, it is assumed that these had wildtype sequence)

Supplementary Figure S6: Sequence data for *APRT* PCR amplicons

The alignment shows sequence data across the gRNA region for chlorate-resistant and 2-fluoroadenine-resistant clones yielding *APRT* PCR amplicons close to the size of the WT amplicon. Base pairs verified by chromatogram inspection are shown as lower case letters. Ambiguous bases are given as "N". The first column indicates the experimental condition used (i.e. growth in Nitrate Medium ("Nit") vs TAP medium ("TAP")) prior to electroporation with Cas9 RiboNuclear Protein (RNP) complexes. Electroporations contained a *NIA1* -directed guide RNA (gRNA; "NIA1_Disrupt; Table 1) and a *NIA1* -directed single stranded oligonucleotide (ssODN; "NIA1_STOP_BclI"; Table 3). The "(+)" designation indicates that these colonies were also derived from electroporations containing an *APRT* -directed gRNA and an *APRT* -directed ssODN. The second column confirms that these clones were resistant to 2-fluoroadenine (suggesting they possess *APRT* mutations) while the third column gives the original colony number from the selection plates. The fourth column shows the amplicon sequence aligned against the WT genomic sequence, and the fifth column includes details of the insertions. Note that the inserted DNA is generally derived from the sequence around the gRNA and ssODN homology region, either from the ssODN or the excised genomic DNA or both, suggesting both indel formation and homology-directed repair (HDR) have been active at the site over the period of CRISPR activity.

Condition	Colony	200bp core NIA1 sequence alignment	Insert/deletion sequence
WT genomic		CTGCCCTCCGTCACCTTCCCGGTGACGCTGGTGTGCGCTGGCAACCGCCGCAAGGAGGAGAACATGCTGAAGAAGAGCATTGGCTTCAACTGGGGCCCTTGTGCCACCAGCACCACCTACTGGACGGGCGTGCGGCTGCGCGACCTGTTGCAGCACGCCGGCATCAAGGTGTGTGTGTGTGTGTGTGTATAGCTGACAG	
Nit(+)	N3	CTGCCCTCCGTCACCTTCCCGGTGACGCTGGTGTGCGCTGGCAACCGCCGCAAGGAGGAGAACATGCTGAAGAAGAGCATTGGCTTCAACTGGGGCCCTTGTGCCACCAGCACCACCTACTGGACGGGCG----------CGACCTGTTGCAGCACGCCGGCATCAAGGTGTGTGTGTGTGTGTGTGTATAGCTGACAG	10 nt deletion
Nit(+)	N9	CTGCCCTCCGTCACCTTCCCGGTGACGCTGGTGTGCGCTGGCAACCGCCGCAAGGAGGAGAACATGCTGAAGAAGAGCATTGGCTTCAACTGGGGCCCTTGTGCCACCAGCACCACCTACTGGACGGGCGTGCGGCTGCGCGACCTGTTGCAGCACGCCGGCATCAAGGTGTGTGTGTGTGTGTGTGTATAGCTGACAG	36 nt insertion (see N9 insertion, below)
Nit(+)	N16	CGTCNCCTTCCCGGTGANGCTGGTGTGCGCTGGCAACCGCCGCAAGGAGGAGAACATGCTGAAGAAGAGCATTGGCTTCAACTGGGGCCCTTGTGCCACCAGCACCACCTACTGATCAGGCGTGCGGCTGCGCGACCTGTTGCAGCACGCCGGCATCAAGGTGTGTGTGTGTGTGTGTGTATAGCTGACAG	Correct insertion of ssODN
Nit(+)	N21	CTGCCCTCCGTCACCTTCCCGGTGACGCTGGTGTGCGCTGGCAACCGCCGCAAGGAGGAGAACATGCTGAAGAAGAGCATTGGCTTCAACTGGGGCCCTTGTGCCACCAGCACCACCTACTGATCAGGCGTGCGGCTGCGCGACCTGTTGCAGCACGCCGGCATCAAGGTGTGTGTGTGTGTGTGTGTATAGCTGACAG	Correct insertion of ssODN
Nit(+)	N47	5' sequence unclear: further mutations can't be ruled out CATGNTGAAGAAGAGCATTGGCNTCAActGgggcCcttgtgcCACCAGCACCACNTNC-[51 nt]-GTGCGGCANCANGTCGNGCAGCAGCACGCCGGCATCAAGGTGTGTGTGTGTGTGTGTGTATAGCTGACAG	9nt deletion with 51nt insertion (see N47 insert, below)
Nit(+)	N51	Possible correct ssODN but lack sequence to confirm TGGGGCCCTTGTGCCACCAGCACCACCTACTGATCAGGCGTGCGGCTGCGCGACCTGTTGCAGCACGCCGGCATCAAGGTGTGTGTGTGTGTGTGTGTATAGCTGACAG	Probably correct insertion of ssODN but insufficient sequence to confirm at present
Nit(+)	N60	CTGCCCTCCGTCACCTTCCCGGTGACGCTGGTGTGCGCTGGCAACCGCCGCAAGGAGGAGAACATGCTG------[69bp ins]----CTGGGGCCCTTGTGCCACCAGCACCACCTACTGATCAGGCGTGCGGCTGCGCGACCTGTTGCAGCACGCCGGCATCAAGGTGTGTGTGTGTGTGTGTGTATAGCTGACAG	21 nt deletion (see N60 insert, below)
Nit(+)	N64	CTGCCCTCCGTCACCTTCCCGGTGACGCTGGTGTGCGCTGGCAACCGCCGCAAGGAGGAGAACATGCTGAAGAAGAGCATTGGCTTCAACTGGGGCCCTTGTGCCACCAGCACCACCTACTGATCAGGCGTGCGGCTGCGCGACCTGTTGCAGCACGCCGGCATCAAGGTGTGTGTGTGTGTGTGTGTATAGCTGACAG	39 nt insertion (see N64 insert, below)
Nit(+)	N74	CTGCCCTCCGTCACCTTCCCGGTGACGCTGGTGTGCGCTGGCAACCGCCGCAAGGAGGAGAACATGCTGAAGAAGAGCATTGGCTTCAACTGGGGCCCTTGTGCCACCAGCACCACCTACTGGACGGGCGTGCGGCTGCGCGACCTGTTGCAGCACGCCGGCATCAAGGTGTGTGTGTGTGTGTGTGTATAGCTGACAG	38 nt insertion (see N74 insert, below)
TAP(+)	T2	CTGCCCTCCGTCACCTTCCCGGTGACGCTGGTGTGCGCTGGCAACCGCCGCAAGGAGGAGAACATGCTGAAGAAGAGCATTGGCTTCAACTGGGGCCCTTGTGCCACCAGCACCACCTACTGATCAGGCGTGCGGCTGCGCGACCTGTTGCAGCACGCCGGCATCAAGGTGTGTGTGTGTGTGTGTGTATAGCTGACAG	Correct insertion of ssODN
TAP(+)	T13	CTGCCCTCCGTCACCTTCCCGGTGACGCTGGTGTGCGCTGGCAACCGCCGCAAGGAGGAGAACATGCTGAAGAAGAGCATTGGCTTCAACTGGGGCCCTTGTGCCACCAGCACCACCTACTGATCAGGCGTGCGGCTGCGCGACCTGTTGCAGCACGCCGGCATCAAGGTGTGTGTGTGTGTGTGTGTATAGCTGACAG	Correct insertion of ssODN
TAP(+)	T50	CTGCCCTCCGTCACCTTCCCGGTGACGCTGGTGTGCGCTGGCAACCGCCGCAAGGAGGAGAACATGCTGAAGAAGAGCATTGGCTTCAACTGGGGCCCTTGTGCCACCAGCACCACCTACTGGACGGGCGTGCGGCTGCGCGACCTGTTGCAGCACGCCGGCATCAAGGTGTGTGTGTGTGTGTGTGTATAGCTGACAG	C insertion at orange nt
TAP(+)	T81	CTGCCCTCCGTCACCTTCCCGGTGACGCTGGTGTGCGCTGGCAACCGCCGCAAGGAGGAGAACATGCTGAAGAAGAGCATTGGCTTCAACTGGGGCCCTTGTGCCACCAGCACCACCTACTGATCAGGCGTGCGGCTGCGCGACCTGTTGCAGCACGCCGGCATCAAGGTGTGTGTGTGTGTGTGTGTATAGCTGACAG	Correct insertion of ssODN
TAP(+)	T87	CTGCCCTCCGTCACcTTCCCGGTGACGCTGGTGTGCGCTGGCAACCGCCGCAAGGAGGAGAACATGCTGAAGAAGAGCATTGGCTTCAAcTGGGGCCCTTGTGCCACCAGCACCACCTACTGgaca-------ggcTGCGCGACCTGTtGCAGCACGCCGGCATCAAggtGTGTGTGTGTGTGTGTGTATAGcTGACAG	A insertion, 8bp deletion
TAP(+)	T91	CTGCCCTCCGTCACCTTCCCGGTGACGCTGGTGTGCGCTGGCAACCGCCGCAAGGAGGAGAACATGCTGAAGAAGAGCATTGGCTTCAACTGGGGCCCTTGTGCCACCAGCACCACCTACTGATCAGGCGTGCGGCTGCGCGACCTGTTGCAGCACGCCGGCATCAAGGTGTGTGTGTGTGTGTGTGTATAGCTGACAG	Correct insertion of ssODN
TAP(+)	T95	CTGCCCTCCGTCACCTTCCCGGTGACGCTGGTGTGCGCTGGCAACCGCCGCAAGGAGGAGAACATGcTGAAGAAGAGCATTGGCTTCAACTGGGGCCCTTGTGCCACCAGCACCACCTACTGATCAGGCGTGCGGCTGCGCGACCTGTTGCAGCACGCCGGCATCAAGGTGTGTGTGTGTGTGTGTGTATAGCTGACAG	Correct insertion of ssODN
Nit(+)	N23	CTGCCCTCCGTCACCTTCCCGGTGACGCTGGTGTGCGCTGGCAACCGCCGCAAGGAGGAGAACATGCTGAAGAAGAGCATTGGCTTCAACTGGGGCCCTTGTGCCACCAGCACCACCTACTGGACGGGCGTGCGGCTGCGCGACCTGTTGCAGCACGCCGGCATCAAGGTGTGTGTGTGTGTGTGTGTATAGCTGACAG	23 nt insertion (see N23 insert, below)
Nit(+)	N35	CTGCCCTCCGTCACCTTCCCGGTGACGCTGGTGTGCGCTGGCAACCGCCGCAAGGAGGAGAACATGCTGAAGAAGAGCATTGGCTTCAACTGGGGCCCTTGTGCCACCAGCACCACCTACTGGACGGGCGTGCGGCTGCGCGACCTGTTGCAGCACGCCGGCATCAAGGTGTGTGTGTGTGTGTGTGTATAGCTGACAG	63 nt insertion (see N35 insert, below)
Nit(+)	N44	CTGCCCTCCGTCACCTTCCCGGTGACGCTGGTGTGCGCTGGCAACCGCCGCAAGGAGGAGAACATGCTGAAGAAGAGCATTGGCTTCAACTGGGGCCCTTGTGCCACCAGCACCACCTACTGGACGGGCCTGCGGCTGCGCGACCTGTTGCAGCACGCCGGCATCAAGGTGTGTGTGTGTGTGTGTGTATAGCTGACAG	G insert at orange nt
TAP(+)	T6	AGGGCCCCAGTTGAAGCCGTGCGGCTGC--GCGACCTGTTGCAGCACGCCGGCATCAAGGTGTGTGTGTGTGTGTGTGTATAGCTGACAG	108bp deletion, separate insertion of unknown length (inversion of ssODN) at 5' end
TAP(+)	T22	CTGCCCTCCGTCACCTTCCCGGTGACGCTGGTGTGCGCTGGCAACCGCCGCAAGGAGGAGAACATGCTGAAGAAGAGCATTGGCTTCAACTGGGGCCCTTGTGCCACCAGCACCACCTACTGATCGGGCGTGCTGCAACAGGTCGCGCAGCCGCACGCCGGCATCAAGGTGTGTGTGTGTGTGTGTGTATAGCTGACAG	Discordant sequence probably inversion of GGCGTGCGGCTGCGCGACCTGTTGCA
TAP(+)	T28	CTGCCCTCCGTCACCTTCCCGGTGACGCTGGTGTGCGCTGGCAACCGCCGCAAGGAGGAGAACATGCTGAAGAAGAGCATTGGCTTCAACTGGGGCCCTTGTGCCACCAGCACCACCTACTGATCAGGCGTGCGGCTGCGCGACCTGTTGCAGCACGCCGGCATCAAGGTGTGTGTGTGTGTGTGTGTATAGCTGACAG	Correct insertion of ssODN

Key: **Dark green** = point mutations; **Orange nt** = site of insertion; Light blue text = insert sequence; Plum text = inversions; dashes = deleted nt, with insert length if deleted nt were replaced by an insert; Ambiguous nt = N
Wt genomic sequence incudes PAM (magenta), gRNA (dark blue) and site of ssODN sequence (light green). Correct insertion of ssODN marked by blue shading.

Sequence of inserts:

Clone	Insert sequence
**N9**	GCCCTTGTGCCACCAGCACCACCTACTGATCAGGCG (inserted at orange nt)
**N47**	ANGNNGGTCATTNNNGGNNCTCAGNNCANANNNNCNTCANNNNGNNCCNNN
**N60**	GCCCATTCCTTGTGCCACCAGCACCACCTACTGATCAGGCGTGCGGCTGCGCGACCTGTTGCAGCACTC insert at site of deletion
**N64**	GACGGGCTGGGGCCCTTGTGCCACCAGCACCACCTACTG (inserted at orange nt)
**N74**	GGCCCTTGTGCCACCAGCACCACCTACTATCGGCGTGG (inserted at orange nt)
**N23**	CCAGCACCACCTACTGATCAGGC
**N35**	CTGAAGAAGAGCATTGGCTTCAACTGGGGCCCTTGTGCCACCAGCACCACCTACTGATCAGGC

Notes concerning clones that were not sequenced:

PCR evidence of insertions and deletions

- No clear amplicon band (i.e. loss of primer site) was observed by PCR for clones N9, N11, N27, N32, N36, N59, N65, N66, N82, N90, T1, T8, T11, T42, T49, T86
- Large amplicon insertions were evidenced by PCR for clones N31, N35, N67, N79, T24, T35, T46, T56, T62, T66, T84
- Large amplicon deletions were evidenced by PCR for clones N37, T43, T16

Supplementary Figure S7: Sequence data for *NIA1* PCR amplicons

The alignment shows sequence data across the gRNA region for chlorate-resistant clones yielding PCR amplicons close to the size of the WT amplicon. Base pairs verified by chromatogram inspection are shown as lower case letters. Ambiguous bases are given as "N". The first column indicates the experimental condition used (growth in Nitrate Medium (Nit) vs TAP medium (TAP) prior to electroporation with Cas9 RiboNuclear Protein (RNP) complexes. Electroporations contained a NIA1-directed gRNA ("NIA1_Disrupt; Table 1) and a NIA1-directed ssODN (NIA1_STOP_BclI; Table 3). The "(+)" designation indicates that these colonies were further derived from electroporations containing an APRT-directed gRNA and an APRT-directed ssODN. The second column gives the original colony number from the selection plates. The third column shows the amplicon sequence aligned against the WT genomic sequence, and the fourth column includes details of the insertions. Note that the inserted DNA is generally derived from the sequence around the gRNA and ssODN homology region, either from the ssODN or the excised genomic DNA or both, suggesting both NHEJ and HDR have been active at the site over the period of CRISPR activity.
